# Cladogenesis and reticulation in *Cuscuta* sect. *Denticulatae* (Convolvulaceae)

**DOI:** 10.1007/s13127-018-0383-5

**Published:** 2018-10-28

**Authors:** Miguel A. García, Saša Stefanović, Catherine Weiner, Magdalena Olszewski, Mihai Costea

**Affiliations:** 1 Department of Biology, University of Toronto Mississauga, Mississauga, ON L5L 1C6 Canada; 2 Royal Botanic Gardens Kew, Richmond, Surrey, TW9 3AE UK; 3 Department of Biology, Wilfrid Laurier University, Waterloo, ON N2L3C5 Canada

**Keywords:** Host shift, Host range, Hybridization, Polyploidy, Parasitic plant, Speciation

## Abstract

As traditionally circumscribed, *Cuscuta* sect. *Denticulatae* is a group of three parasitic plant species native to the deserts of Western USA (*Cuscuta denticulata*, *Cuscuta nevadensis*) and the central region of Baja California, Mexico (*Cuscuta veatchii*). Molecular phylogenetic studies confirmed the monophyly of this group and suggested that the disjunct *C. veatchii* is a hybrid between the other two species. However, the limited sampling left the possibility of alternative biological and methodological explanations. We expanded our sampling to multiple individuals of all the species collected from across their entire geographical ranges. Sequence data from the nuclear and plastid regions were used to reconstruct the phylogeny and find out if the topological conflict was maintained. We obtained karyotype information from multiple individuals, investigated the morphological variation of the group thorough morphometric analyses, and compiled data on ecology, host range, and geographical distribution. Our results confirmed that *C. veatchii* is an allotetraploid. Furthermore, we found previously unknown autotetraploid population of *C. denticulata*, and we describe a new hybrid species, *Cuscuta psorothamnensis*. We suggest that this newly discovered natural hybrid is resulting from an independent (and probably more recent) hybridization event between the same diploid parental species as those of *C. veatchii*. All the polyploids showed host shift associated with hybridization and/or polyploidy and are found growing on hosts that are rarely or never frequented by their diploid progenitors. The great potential of this group as a model to study host shift in parasitic plants associated with recurrent allopolyploidy is discussed.

**Electronic supplementary material:**

The online version of this article (10.1007/s13127-018-0383-5) contains supplementary material, which is available to authorized users.

## Introduction

*Cuscuta* (dodders) is a plant genus of nearly 200 species of stem parasites (Yuncker 1932; García et al. 2014; Costea et al. 2015a) that has evolved within Convolvulaceae (reviewed by Stefanović and Olmstead 2004, 2005). The genus is nearly cosmopolitan but the majority of species are native to North and South America, belonging to *Cuscuta* subg. *Grammica*, the largest infrageneric group that includes more than 150 species (Costea et al. 2015a). Dodders occur in a great variety of habitats, from temperate to tropical, desert to riparian, littoral to high mountains, grasslands, forests, saline, and disturbed habitats. Similarly to other parasitic plants, dodders act as keystone species in their ecosystems (Press and Phoenix 2005; Graffis and Kneitel 2015). Approximately 15–20 *Cuscuta* spp. worldwide are agricultural and horticultural pests (Dawson et al. 1994; Costea and Tardif 2006), and in most countries, control and quarantine measures target the genus as a whole, ignoring the fact that some species may be endangered or even threatened with extinction (Costea and Stefanović 2009).

Stefanović et al. (2007) noted several cases of conflict between plastid- and nuclear-derived phylogenies indicative of possible reticulation in *Cuscuta* subg. *Grammica*. To further investigate the origin of these conflicts, Stefanović and Costea (2008) expanded their *trnL-F* and nrITS matrices through addition of multiple sequences from 105 species from this subgenus and found five cases of species with a probable hybrid origin. A series of statistical tests by Stefanović and Costea (2008) showed that alternative hypotheses to explain discordant gene topologies, such as incomplete lineage sorting, undetected paralogy, or horizontal gene transfer, were not supported. All these cases in subg. *Grammica* were confirmed and three additional ones were detected using *rbcL* and nrLSU sequences in a broader phylogenetic context with representatives of the entire genus (García et al. 2014). Several cases of topological incongruence were also found for *Cuscuta* subg. *Cuscuta* by García and Martín (2007), but the origin of the conflicts was not further investigated. Costea and Stefanović (2010) discovered that at least four independent hybridization events had occurred in the evolution of *Cuscuta* sect. *Umbellatae* (subg. *Grammica*), two more for this section than previously detected. More recently, a detailed study by Costea et al. (2015b) on *Cuscuta* sect. *Cleistogrammica* (subg. *Grammica*) showed that the worldwide invasive weed *Cuscuta campestris* Yunck (1932) has two divergent groups of nrITS ribotypes. Both of these disparate ribotypes are topologically incongruent with the plastid *trnL-F* phylogeny, in aggregate suggesting the hybrid origin of *C. campestris* (Costea et al. 2015b).

**Fig. 1:**
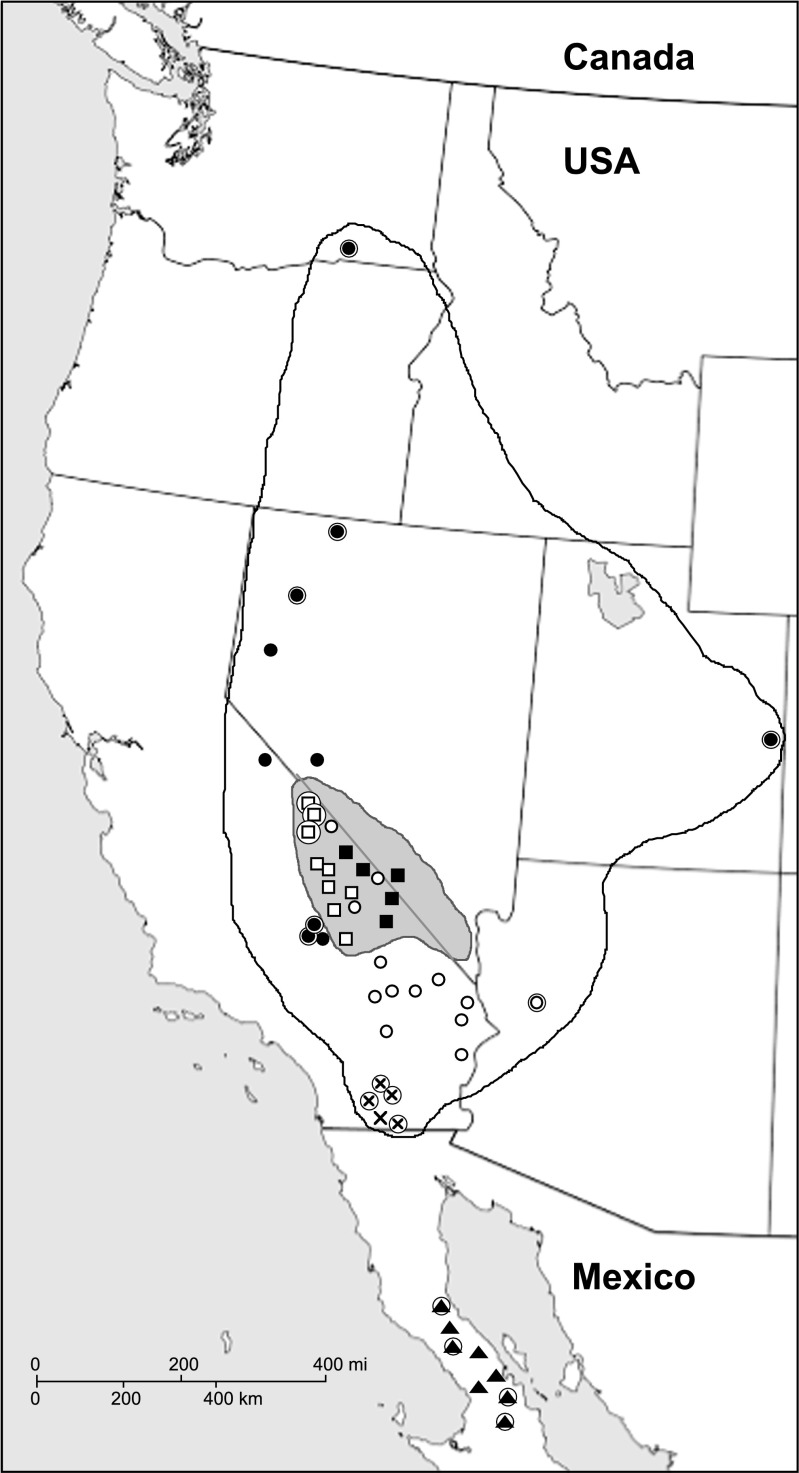
Distribution of *Cuscuta* sect. *Denticulatae* species across their geographic ranges in western North America. Potential extent of distribution for *C. denticulata* is outlined and that of *C. nevadensis* is shaded. Approximate positions of sampling sites used in this study are indicated (for details, see Appendix 1). Circles (solid and open) represent sampling sites for populations of *C. denticulata*, squares (solid and open) represent those of *C. nevadensis*, triangles those of *C. veatchii*, while X symbols stand for the newly described species, *C. psorothamnensis*. Encircled symbols represent material obtained from herbaria; all others are sampled directly in the field, including multiple individuals per population. Solid and open symbols correspond to different haplo- and ribotypes of *C. denticulata* and *C. nevadensis* (see text for details)

Another striking case of topological discordance was found within *Cuscuta* sect. *Denticulatae* (Stefanović and Costea 2008; García et al. 2014). This group of species in subg. *Grammica* is well characterized morphologically by the radicular end of the embryo spherically enlarged in a ball-like structure that increases in volume during seed maturation (Costea et al. 2015a). Such a feature is not present in any other clade of *Cuscuta* and this synapomorphy is thought to be an adaptation for seed germination on the host while the fruit is still enclosed by the perianth (vivipary). The enlarged embryo probably stores nutrients and water as an adaptation to germination in desert environments (Costea et al. 2005). Section *Denticulatae* includes three species distributed in Western USA (*Cuscuta denticulata* Engelm.; *Cuscuta nevadensis* I.M.Johnst.) and the Central Desert of Baja California in Mexico (*Cuscuta veatchii* Brandegee) (Fig. 1). Of the three species, *C. denticulata* has the broadest geographical distribution and host preference, whereas *C. nevadensis* has narrower geographical and host ranges, occurring sympatrically with *C. denticulata* (Costea et al. 2005). *Cuscuta veatchii* has a disjunct distribution and grows only on *Pachycormus discolor* (Benth.) Coville (Anacardiaceae). The phylogenetic analyses by Stefanović and Costea (2008) placed *C. veatchii* in a clade with *C. denticulata* on the nrITS tree, whereas this species was resolved with *C. nevadensis* on the plastid *trnL-F* tree, in both cases with 100% bootstrap support. The authors concluded that this topological incongruence, together with the life history, ecological, and biogeographical data, was consistent with a hybrid origin of *C. veatchii*. However, some alternative topology tests failed to find statistical difference between optimal and constrained trees among plastid and nuclear data. Also, in that study, the sampling was limited to two to three individuals per species, and because the clade included only three taxa and a root, a topological distortion such a nearest-neighbor interchange (NNI) could not be ruled out, leaving ancestral polymorphism or incomplete lineage sorting as viable alternative explanations for the discordance between nuclear and plastid trees.

In the present study, we further investigate the hybridization hypothesis for the origin of *C. veatchii* by expanding our sampling to multiple individuals for all species involved from their entire geographical distribution as well as by obtaining additional critically needed corroborating information. Thus, the objectives of this study are (1) to analyze new nrITS and *trnL-F* sequence data to further investigate if the initial topological conflict is maintained with the expanded sampling, (2) obtain for the first time in this group information about chromosome numbers from multiple individuals of all species involved to find out if karyotype data support the hybridization hypothesis, (3) investigate the morphological variation of the group thorough explicit morphometric analyses, and (4) compile additional data about ecology, host range, and geographical distribution using both herbarium and newly collected specimens for this study. We also describe and illustrate one new allotetraploid species that belongs to this clade/section.

## Materials and methods

### Taxon sampling

As part of our overall efforts to elucidate evolutionary history and biology of *Cuscuta*, and in preparation for a future monograph of the genus, we have searched for relevant specimens in over 100 herbaria over the years. Approximately 400 herbarium specimens were identified, annotated, and examined for basic morphology, as well as host and geographical range for species in *Cuscuta* sect. *Denticulatae*. In addition, we conducted a series of targeted field trips to the areas of particular interest for this section in the springs/summers of 2013–2016. Efforts were made to ensure a sampling from localities across the entire known geographical range for each of the species (Costea et al. 2005).

From these collections, a total of 90 specimens were selected for the morphometric analyses: 37 for *C. denticulata*, 25 for *C. nevadensis*, 22 for *C. veatchii*, and 6 for the new species described here, *Cuscuta psorothamnensis* (Appendix 1).

Compared to our previous studies (Stefanović et al. 2007; Stefanović and Costea 2008; García et al. 2014), we substantially improved our population-level sampling across all species of *Cuscuta* sect. *Denticulatae*, representing the breadth of their morphological diversity and geographical range (Fig. 1). In addition to the eight DNA samples used previously, total genomic DNA was isolated from 41 newly obtained localities, coming from additional herbarium material and field-collected (Appendix 1). Herbarium-derived samples represented a single individual per population (samples circled in Fig. 1). To account for both intra-specific and potential intra-population sequence variation of DNA markers, two to five individuals were sampled per locality in our field trips. Within a locality, multiple specimens were collected growing on separate host plants (> 1 m apart from each other). Despite the host separation, some of the specimens still could be a product of multiple individual seedlings infecting a plant (Costea and Tardif 2006), and we considered these to be “bulked” individuals. Therefore, when available, for a number of samples, we collected seed as well. After scarification in concentrated sulfuric acid for 2–5 min and multiple rinses in distilled water, we germinated seed on wet filter paper in Petri dish. DNA was extracted from individual seedlings as well as a mix of seedlings, to increase the overall amounts of DNA (Appendix 1). On the phylogenetic trees, uppercase letters after the DNA accession number indicate individuals growing on different host plants in natural populations, whereas lowercase letters indicate individual seedlings from the same mother plant. A total of 97 ingroup samples were used for the molecular analyses in this study. Based on our previous, more inclusive phylogenetic analyses of *Cuscuta* subg. *Grammica* (Stefanović et al. 2007; Stefanović and Costea 2008), and the whole genus (García et al. 2014), *Cuscuta compacta* Juss. ex Choisy, a species belonging to the sister section *Oxycarpae*, was selected as the outgroup.

A subset of 24 samples was also used for chromosome counts: 10 of *C. denticulata*, 7 of *C. nevadensis*, 3 of *C. psorothamnensis*, and 4 of *C. veatchii* (Appendix 1).

### Molecular techniques

Sequences for the internal transcribed spacer (ITS) region of nuclear ribosomal DNA (nrDNA) as well as *trnL-F* intron/spacer region from the plastid genome (ptDNA) were obtained to infer phylogenetic relationships among species of sect. *Denticulatae*. DNA extractions, polymerase chain reaction (PCR) reagents and conditions, and amplicon purifications followed the protocols detailed in Stefanović et al. (2007). Cleaned products were sequenced at the McGill University and Génome Québec Innovation Centre (Canada). By direct sequencing of nrITS amplicons using Sanger sequencing, significant amounts of additive polymorphic sites were detected for specimens collected from the Anza-Borrego Desert State Park, CA, USA, originally identified as *C. denticulata*, but henceforth referred to in this study as *Cuscuta psorothamnesis*. Purified PCR products were cloned for all the species using the pGEM-T Easy Vector II cloning kit (Promega) and multiple clones per individual were sequenced. Cloning of nrITS amplicons was performed also for one or two individuals of all the species even when no polymorphisms were detected by direct sequencing. To reduce uninformative repetition in both nrITS and *trnL-F* matrices, and to reduce the computational burden, all the individuals from the same locality that had identical sequences were grouped into a single operational taxonomic unit (OTU). In this fashion, including the outgroup, a total of 115 nrITS and 51 *trnL-F* sequences were analyzed (Table 1). New sequences generated for this study (106 nrITS and 45 *trnL-F*) were deposited in GenBank (accession numbers MH923079–MH923184 for ITS and MH920261–MH920306 for *trnL-F*; see Appendix 1). Sequences from the outgroup species, *C. compacta*, were newly obtained for this paper and also submitted to GenBank with numbers MH920312 (nrITS) and MH920261 (*trnL-F*).

**Table 1:** Summary descriptions for sequences included in phylogenetic analyses and trees derived from individual and combined datasets of *Cuscuta* sect. *Denticulatae*

	Nuclear (ITS)	Plastid (*trnL-F*)	Combined
Number of OTUs included	115	51	38
Sequence characteristics			
Aligned length	670	547	1215
Number of indels coded	3	8	11
Variable sites	250	89	188
Parsimony informative sites	93	52	98
MP tree characteristics			
Length	383	108	231
CI/RI	0.802/0.964	0.935/0.990	0.931/0.988
Bayesian analyses			
Model of DNA evolution	HKY + G	GTR + G	HKY + G/GTR + G
Mean *-lnL*	3299.63	1294.32	2759.57

*CI* consistency index, *RI* retention index

### Sequence alignment and phylogenetic analyses

Sequencher 4.2 (Gene Codes Corp., Ann Arbor, MI, USA) was used to assemble and edit chromatograms of complementary strands. Sequences were aligned manually using Se-Al v.2.0a11 (Rambaut 2002). Gaps were manually coded as simple indels (Simmons and Ochoterena 2000) and appended to the sequence matrices as binary characters. Three gaps were coded for the nrITS matrix and eight for the *trnL-F* matrix, whereas all the gaps were also included in the combined matrix. Phylogenetic analyses were conducted under parsimony and Bayesian optimality criteria; summary descriptions of these analyses, for individual as well as combined datasets, are provided in Table 1.

Under parsimony criterion, nucleotide characters were treated as unordered and all changes were equally weighted, except for the indel partition which was double weighted. Searches for most parsimonious (MP) trees for all the matrices were performed using a two-stage strategy using PAUP* v.4.0a147 (Swofford 2002). First, the analyses involved 10,000 replicates with stepwise random taxon addition, tree bisection-reconnection (TBR) branch swapping saving no more than 10 trees per replicate, and MULTREES off. The second round of analyses was performed on all trees in memory with the same settings except with MULTREES on and MaxTrees set to one million. Support for clades was inferred by nonparametric bootstrapping (Felsenstein 1985), using 500 heuristic bootstrap replicates, each with 20 random addition cycles, TBR branch swapping, and MULTREES option off (DeBry and Olmstead 2000). Nodes receiving bootstrap (BS) values < 60, 60–75, and > 75% were considered weakly, moderately, and strongly supported, respectively.

Bayesian phylogenetic inferences were performed using MrBayes v.3.2.6 (Ronquist et al. 2012) run on the CIPRES Science Gateway (Miller et al.  2010). The program MrModeltest v.2.3 (Nylander 2004) was used to determine the model of sequence evolution for each dataset by the Hierarchical Likelihood Ratio Tests (hLRTs) and the Akaike Information Criterion (AIC). For the sequence partition of the nrITS matrix, the Hasegawa-Kishino-Yano model of DNA substitution (Hasegawa et al. 1985) with addition of rate variation among nucleotides following a discrete gamma distribution (HKY + G) was selected as the best fit. For the *trnL-F* matrix, the General Time Reversible model (Tavaré 1986) with addition of rate variation among nucleotides following a discrete gamma distribution (GTR + G) was the model chosen; see Table 1 for details. In all cases, the restriction 0/1 state model was selected for the indel partitions. Each Bayesian analysis consisted of two runs, each for 10 million generations starting from a random tree using the default priors, and eight Markov chains sampled every 5000 generations. Of the trees obtained from the two runs, the first 25% were discarded as burn-in. In all analyses, the standard deviation of split frequencies was below 0.01 as indication of convergence. The 50% majority-rule consensus trees and the Bayesian posterior probabilities (PP) were obtained in MrBayes from the 3002 remaining trees. Only the nodes receiving ≥ 0.95 PP were considered statistically significantly supported (Rannala and Yang 1996).

To conduct a total-evidence analysis, a matrix of concatenated nrITS, *trnL-F*, and indel partitions was created for the individuals and populations of the putative parental species (*C. denticulata* and *C. nevadensis*) but excluding *C. veatchii* and the individuals of *C. psorothamnensis*. This matrix was analyzed using both parsimony and Bayesian inferences following the same procedures than for individual datasets (see Table 1).

### Chromosome techniques

Flower buds were collected in the field (Appendix 1) and fixed in Carnoy’s solution (absolute ethanol/glacial acetic acid, 3:1, *v*/*v*), stored at − 20 °C, stained in darkness with 4% Wittman’s hematoxylin for at least 24 h, and mounted and squashed in 45% acetic acid. Alternatively, stems, seedlings, or flower meristems (grown from seed in the University of Toronto Mississauga greenhouse) were prefixed in 8-hydroxyquinoline 0.002 M for 24 h at 10 °C, fixed in Carnoy’s solution for 24 h at room temperature, hydrolyzed in HCl 5 N for 20 min, washed in distilled water, and squashed and mounted in 45% acetic acid. The slides were frozen in liquid nitrogen to remove the coverslip, air-dried and chromosomes stained with 1% hematoxylin for a few seconds, washed with distilled water, air-dried, and permanent mounts prepared in Canada balsam.

### Morphometric analyses

Four OTUs corresponding to *C. denticulata*, *C. nevadensis*, *C. veatchii*, and *C. psorothamnensis* were included in the morphometric analyses to test their morphological distinctiveness. Samples collected in the field (also used for molecular analyses; Appendix 1) were stored in 50% ethanol prior to their use for morphological measurements. Flowers removed from herbarium specimens were steeped in gradually warmed 50% ethanol, which was then allowed to boil for a few seconds to rehydrate tissues. A previous morphological study of sect. *Denticulatae* (Costea et al. 2005) and morphometric studies of other groups within subg. *Grammica* (e.g., sect. *Californicae*, Costea et al. 2009; sect. *Cleistogrammica*, Costea et al. 2015b) provided a preliminary list of useful characters. These characters were further refined using some recent studies on character evolution of gynoecium and perianth (Wright et al. 2011, 2012) and infrastaminal scales (Riviere et al. 2013). Pollen length and width were also measured in all the specimens to determine if a relationship exists between pollen size and chromosome number. In total, 32 characters, 29 continuous and three binary, were used in the morphometric analysis (Appendix 2).

For basic morphology, flowers were dissected under a Nikon SMZ1500 stereomicroscope and imaged with PaxCam Arc digital camera (MIS Inc. 2017, Villa Park, IL) equipped with a Pax-it 8 imaging software. For scanning electron microscopy (SEM) of pollen, we used hexamethydisilazane (HMDS) as an alternative for critical dry point (Costea et al. 2011a, b), and the examination was done at 10 kV using a Hitachi SU1510 variable pressure scanning electron microscope. To determine the extent of morphological variation, the data were visualized with both clustering and ordination methods using PAST (version 3.15; Hammer et al. 2001). Principal Coordinate Analysis (PCoA or Metric Multidimensional Scaling) and Unweighted Pair-Group Average (UPGMA) were both conducted using the Gower’s coefficient of similarity.

### Hosts and geographical range

The geographical distribution, phenology, elevation, and host ranges are based on observations made in the field and from the following herbaria: ARIZ, ASU, BRIT, CAS, CHSC, CIIDIR, CIMI, DS, F, GH, IEB, JEPS, K, MEXU, MICH, MO, NY, RSA, SD, TRTE, UC, US, UCR, and WLU. Host ranges of the four species (OTUs defined as for morphometric studies) were initially analyzed with PCoA which indicated strongly divergent patterns among the four OTUs (results not shown). The host range was visualized as a bipartite network in Fig. 5 and summarized in Suppl. Table S1.

## Results

### Phylogenetic analyses

**Fig. 2:**
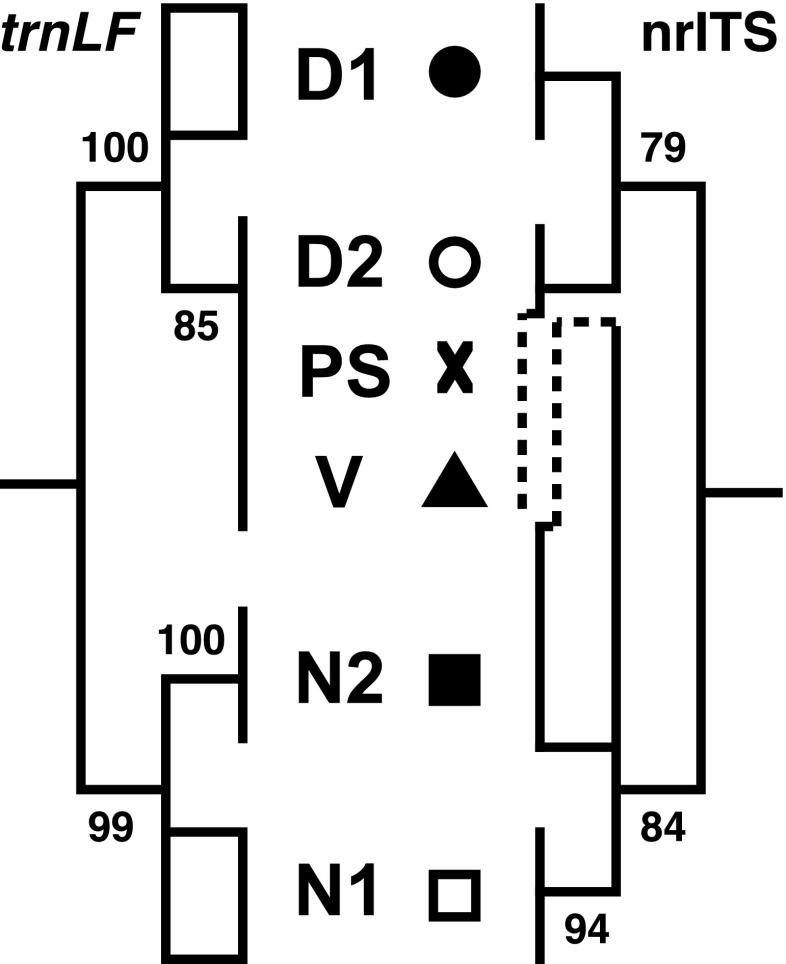
Schematic overview of the phylogenetic relationships in *Cuscuta* sect. *Denticulatae* derived from plastid (*trnL-F*) and nuclear (nrITS) sequence data. For simplicity, only the moderately to strongly supported backbone nodes are shown as resolved. Unresolved groups are represented with a box symbol. For full details, compare with Suppl. Figs. S1 and S2. Symbols are the same as described in Fig. 1

Summary descriptions for sequences obtained from nrITS and *trnL-F* regions are presented in Table 1. Overall phylogenetic relationships in sect. *Denticulatae* are summarized in Fig. 2 (compare with Suppl. Figs. S1 and S2 for details).

Direct sequencing of nrITS amplicons from most of the individuals of the three traditionally accepted species showed no intra-individual sequence variation or clear polymorphisms were limited to only a few positions. This reduced intra-individual nrITS variation was confirmed by cloning in which the number of variable sites ranged from 0 to 8 in *C. denticulata*, 0 to 13 in *C. nevadensis* and 0 to 5 in *C. veatchii*. However, the individuals of what we originally identified as *C. denticulata* but we now treat and describe here as a new species, *C. psorothamnensis*, showed a comparatively very high number of variable sites. Cloning of these amplicons confirmed a high intra-individual variation of nrITS sequences ranging from 2 to 52 nucleotide positions. Phylogenetic analyses of the nrITS sequences resolved two strongly supported clades, labeled here as D and N (Suppl. Fig. S1). One of them, the D clade (BS = 79; PP = 0.94), contained all the sequences of *C. denticulata*, including those found to be autotetraploid (accession number 1398; multiple clones from multiple individuals collected at this locality), as well as a subset of clones obtained from *C. psorothamnensis*. The rest of the clones from the same specimens of this species were resolved in the N clade (BS = 84; PP = 1.00) together with *C. nevadensis* and *C. veatchii*. All the individuals of *C. psorothamnensis* had at least one clone resolved in each of the two major clades. The clone sequences of these individuals had longer branches, especially those found in the clade of *C. nevadensis* (Suppl. Fig. S1). While these two major clades (D and N) were strongly supported, internal resolution within them was very low. For the group of *C. denticulata*, two sub-clades were recovered only under Bayesian analyses but lacking any significant support, whereas a polytomy was recovered under parsimony. Only one sub-clade, including four accessions of *C. denticulata* [165, 485, 1144, and 1473], was recovered by both analyses with strong support (BS = 94; PP = 1.00). As for the group of *C. nevadensis*, two clone sequences from *C. psorothamnensis* were successively sister to a polytomy with the rest of the sequences in which a clade with eight accessions of *C. nevadensis* [476, 585, 1145, 1399, 1409, 1427, 1429, 1480] was recovered with strong support in both analyses (BS = 94; PP = 1.00).

Parsimony and Bayesian analyses of the *trnL-F* matrix resulted in more resolved and internally better supported trees (Suppl. Fig. S2). Two major clades, D and N, were recovered again, but this time with very high support (BS = 100, PP = 1.00 and BS = 99, PP = 1.00, respectively). The D clade contains all the accessions of *C. denticulata* (including autotetraploid 1398), *C. veatchii*, and *C. psorothamnensis*. The sequences of these two species together with those of individuals of *C. denticulata* collected predominantly from its southern parts of distribution (open circles; Fig. 1) are resolved in a moderately to strongly supported internal clade (BS = 85; PP = 1.00). The rest of the sequences, including the four accessions that formed a strongly supported clade on the ITS tree, are not resolved as a lineage here. The N clade includes only sequences of *C. nevadensis*. Within this clade, the eight accessions that were resolved as monophyletic on the ITS tree are not resolved as such on the *trnL-F* trees. The rest of the accessions, however, are on the plastid trees strongly supported as monophyletic (BS = 100; PP = 1.00). This clade includes individuals collected in the eastern parts of distribution of this species (Death Valley, along the border between California and Nevada; eastern parts of Inyo Co., CA, and western parts of Nye Co., NV; solid squares; Fig. 1).

The combined analyses of the matrix, excluding *C. veatchii* and *C. psorothamnesis*, recovered trees with BS = 100 and PP = 1.00 support for the two main clades, D and N (Suppl. Fig. S3). Within the N clade, the lineages recovered previously with either ITS or *trnL-F* individual matrices are here recovered also but with high support. The sub-clade with eight accessions recovered with ITS, in the combined analyses received BS = 86 and PP = 0.99, and corresponds with populations subsequently called as N1 (represented with open squares; Fig. 1). The rest of the accessions of *C. nevadensis* were resolved in a second sub-clade (BS = 86; PP = 0.99), called N2 (represented with solid squares; Fig. 1). Within the D clade, the accessions of *C. denticulata* recovered as monophyletic on the *trnL-F* trees are in the combined analyses also resolved in a sub-clade with high support (BS = 96; PP = 1.00). These accessions are called D2 (represented with open circles; Fig. 1). The remaining accessions of *C. denticulata* are called D1 (represented with solid circles; Fig. 1).

### Karyotypes

**Fig. 3:**
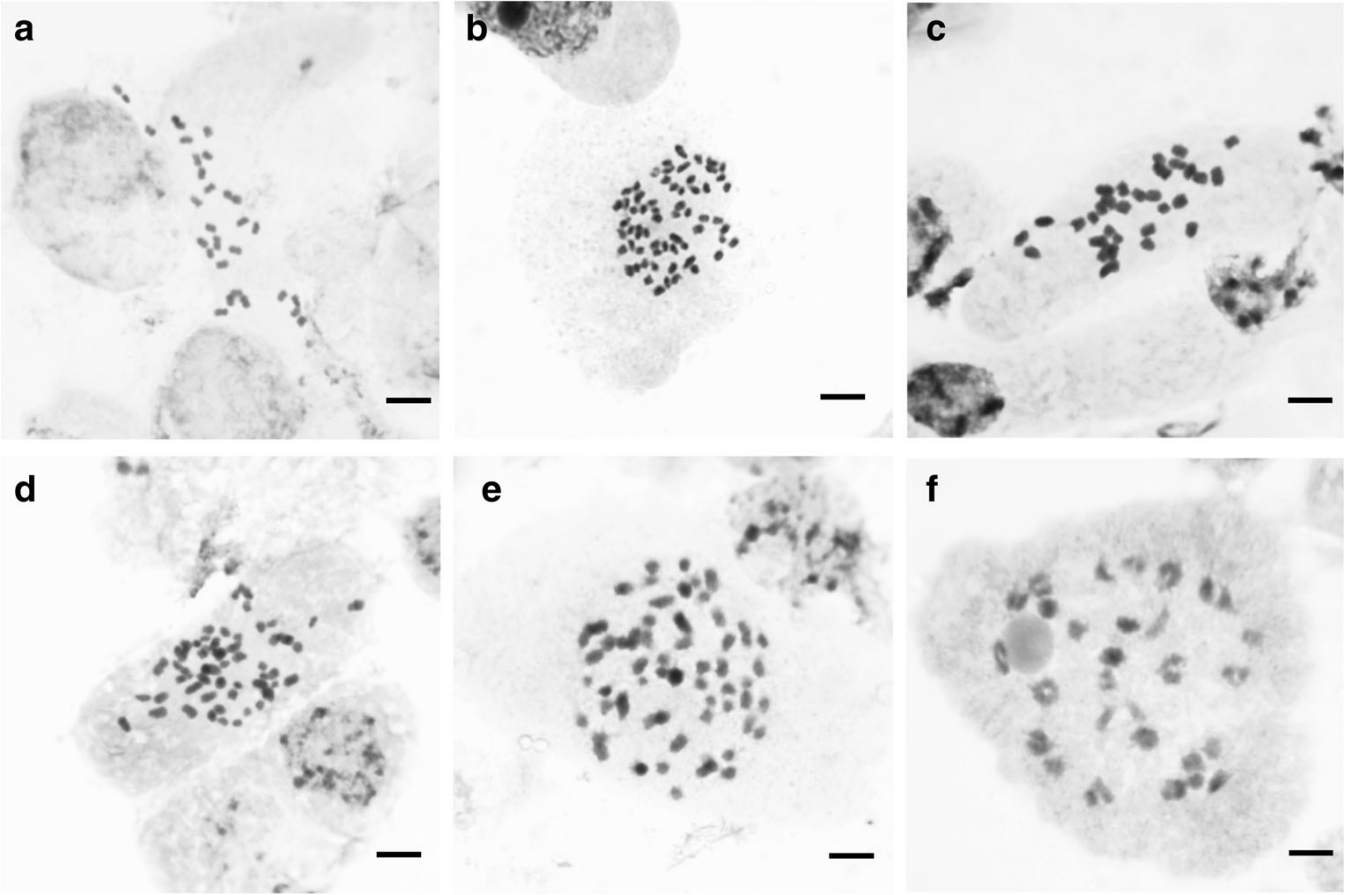
Karyology of *Cuscuta* sect. *Denticulatae*. Mitotic metaphases and interphase nuclei of **a** diploid *C. denticulata* (2*n* = 30), **b** tetraploid *C. denticulata* (2*n* = 60), **c** *C. nevadensis* (2*n* = 30), **d** *C. veatchii* (2*n* = 60), and **e** *C. psorothamnensis* (2*n* = 60). **f** Diakinesis of *C. veatchii* showing *n* = 30 bivalents. Note the scale bar is the same across and corresponds to 5 μm

Chromosome counts are listed in Appendix 1. Individuals of *C. denticulata* are generally diploids with 2*n* = 30 monocentric chromosomes and symmetrical karyotype (Fig. 3a). Interphase nuclei are reticulate, with a few small chromocenters. One individual of this species (DNA accession number 1398) from the Walker Pass, Kern Co., CA, is a tetraploid with 2*n* = 60 chromosomes (Fig. 3b). Chromosome morphology and size of this individual were similar to those of *C. denticulata* diploids. This individual showed morphological features similar to other specimens of *C. denticulata* but with larger flower parts (see section on morphological characters). For some specimens of *C. nevadensis*, chromosomes were not easy to observe, and counts were between 2*n* = 28 and 2*n* = 30. Nevertheless, when good metaphases were found, we always counted 30 chromosomes. Based on this, we consider *C. nevadensis* to be a diploid with 2*n* = 30. Chromosomes of *C. nevadensis* are monocentric, the karyotype is symmetrical, and the interphase nuclei are areticulate, exhibiting large and well-defined chromocenters (Fig. 3c). Also, chromosomes in this species are larger than those of *C. denticulata* (Fig. 3c; note that all images in this panel are shown at the same magnification).

All the individuals of *C. veatchii* sampled are tetraploids with 2*n* = 60 chromosomes (Fig. 3d). Whereas in the diploids all the chromosomes were of similar size, *C. veatchii* has metaphase chromosomes with a mix of different sizes. Interphase nuclei in this species have a mixture of small and well-delineated chromocenters. The three individuals of *C. psorothamnensis* included in our karyological studies were also tetraploids (2*n* = 60; Appendix 1) with similar karyotype and interphase nuclei to *C. veatchii* (Fig. 3e). In both tetraploid species, meiosis was regular with *n* = 30 bivalents (Fig. 3f).

### Morphometric analyses

**Fig. 4:**
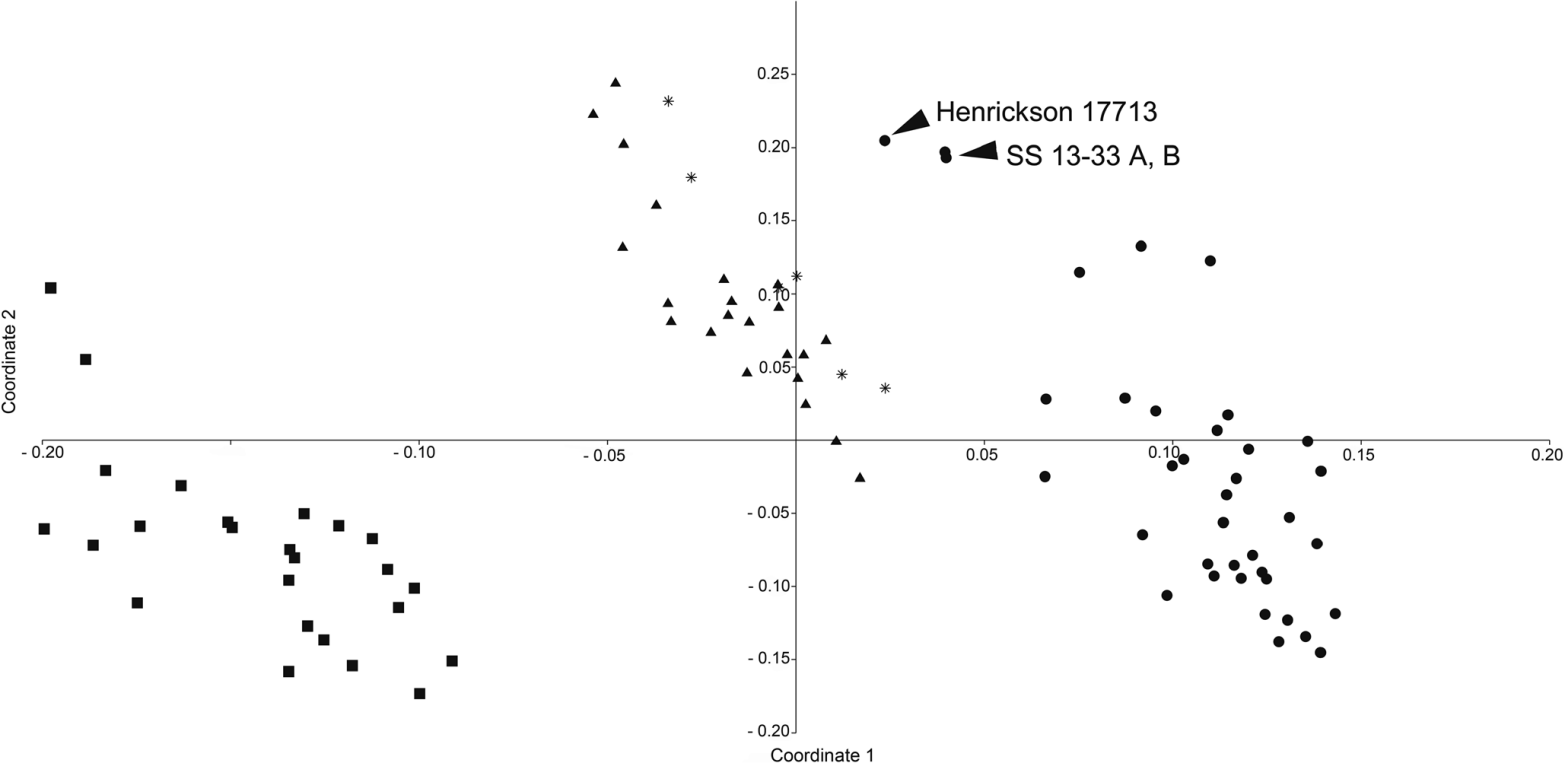
Principal Coordinate Analysis (PCoA) using all morphological characters (Appendix 2). The first coordinate axis (62.621% of the variance) separated *C. nevadensis* from *C. denticulata* and *C. veatchii* together with *C. psorothamnesis*. The second coordinate axis (9.297% of the variance) clearly separated *C. denticulata* from *C. veatchii/C. psorothamnesis* mix. Circles = *C. denticulata*; squares = *C. nevadensis*; triangles = *C. veatchii*; stars = *C. psorothamnensis*. Head arrows indicate the proximity of *SS-13-33* (2*n* = 60) and *Henrickson 17713* (presumed autotetraploid)

With or without the pollen size included in the analyses, PCoA produced three distinct groups: one that corresponded to *C. denticulata*, one for *C. nevadensis*, and one for overlapping clusters of *C. veatchii* and *C. psorothamnensis* (Fig. 4). The first coordinate axis (62.621% of the variance) separated *C. nevadensis* from *C. denticulata* and *C. veatchii/C. psorothamnesis* mix. The second coordinate axis (9.297% of the variance) clearly separated *C. denticulata* from *C. veatchii* and *C. psorothamnesis*. Two specimens of *C. denticulata*—*Stefanović SS-13-33* A and B (Kern Co., CA) and *Henrickson 17713* (Inyo Co., CA)—diverged from their species group because of their larger flowers. Field-collected samples (*Stefanović SS-13-33* A and B) have been determined to be autopolyploids (see “Chromosome techniques” section), but no chromosome data is available for herbarium collection *Henrickson 17713*.

The dendrogram obtained from the UPGMA cluster analysis revealed also three distinct clusters which had a similar composition to the major groups obtained through PCoA analysis: one cluster that contained all *C. denticulata* (including autotetraploids), one that comprised both *C. veatchii* and *C. psorothamnensis*, and one for *C. nevadensis* (Suppl. Fig. 4). The six specimens attributed to *C. psorothamnesis* formed two sub-clusters within *C. veatchii* (Suppl. Fig. 4). Within each species, in general, samples from the same geographical areas did not cluster together. The cophenetic correlation coefficient was 0.8438.

Analyzed independently, pollen data did not permit the separation of any of the four OTUs when using either ordination or clustering methods (results not shown), but some trends were observed. In general, *C. veatchii* has the largest pollen grains, while *C. nevadensis* the smallest. However, there is a significant overlap of the pollen size especially between *C. denticulata* and *C. nevadensis*. The known autopolyploid of *C. denticulata* (*Stefanović SS-13-33*; Kern Co., CA) as well as suspected autopolyploid (*Henrickson 17713*; Inyo Co., CA) have large pollen grains, similar in size to those of *C. veatchii* (results not shown). *C. psorothamnensis* grains were either similar to *C. veatchii* or they were mixed with those of *C. denticulata* and *C. nevadensis*. The shape of pollen grains varied within each OTU from sub-sphaeroidal to prolate. The ploidy level does not affect the number of colpi as all species/accessions have 3(-4)-zonocolpate pollen. Morphology of the tectum is also similar among OTUs: imperforate or with a few isolated punctae, sexine scabrate with isolated granule.

### Host range

**Fig. 5:**
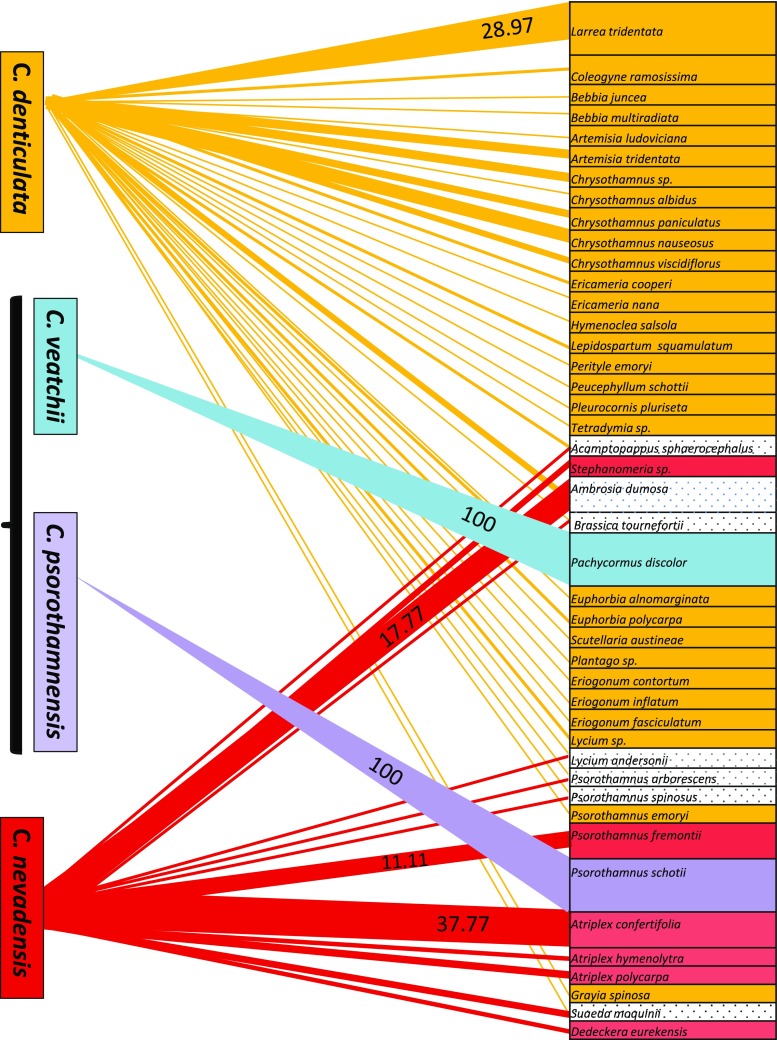
Host ranges of *Cuscuta* sect. *Denticulatae* species visualized as a bipartite network. Four *Cuscuta* species nodes on the left are connected with the corresponding nodes of their hosts on the right. Thickness of the lines representing the edges of the network indicates the frequency of the parasite-host association. Frequencies higher than 10% are indicated in the graph while the rest of the host frequencies are available in Suppl. Table S1. Hosts indicated with gray dots on white are shared between *C. denticulata* and *C. nevadensis*. Note the 100% host specificity of *C. veatchii* and *C. psorothamnensis*

Host ranges of *Denticulatae* species are largely distinct from one another (Fig. 5 and Suppl. Table S1). *Cuscuta denticulata* parasitizes the largest number of species, 35 from 12 families while *C. nevadensis grows* on 15 species from 6 families. Favorite hosts for *C. denticulata* appear to be *Larrea tridentata* J.M.Coult. (Zygophyllaceae) (28.97% frequency) and various species of *Chrysothamnus* (Asteraceae (totaling 27.08% frequency). For *C. nevadensis*, *Atriplex confertifolia* S.Watson (Chenopodiaceae) (37.77%), *Ambrosia dumosa* (A.Grey) W.W.Payne (Asteraceae) (17.67%), and *Psorothamnus fremontii* (A.Grey) Barneby (Fabaceae) (11.11%). The host ranges of *C. denticulata* and *C. nevadensis* overlap with low frequency on six hosts from five families and their most commonly shared host is *A. dumosa* (3.73% for *C. denticulata* and 17.67% for *C. nevadensis*). However, neither in the field nor among herbarium specimens have we ever encountered any two of these species growing together on the same host plant. Most notably, the two hybrid allopolyploid species, *C. veatchii* and *C. psorothamnensis*, are highly host-specific, parasitizing exclusively on *Pachycormus discolor* and *Psorothamnus schottii* (Thor.) Barneby, respectively. Genus *Psorothamnus* is a common host for *C. denticulata*, *C. nevadensis*, and *C. psorothamnensis*, but while the latter grows only on *P. schottii*, the two former may both parasitize *Psorothamnus arborescens* (A.Grey) Barneby and *Psorothamnus spinosus* (A.Grey) Barneby and have never been observed on *P. schottii* (Fig. 5 and Suppl. Table S1).

## Discussion

### Natural hybridization in *Cuscuta* sect. *Denticulatae*

**Fig. 6:**
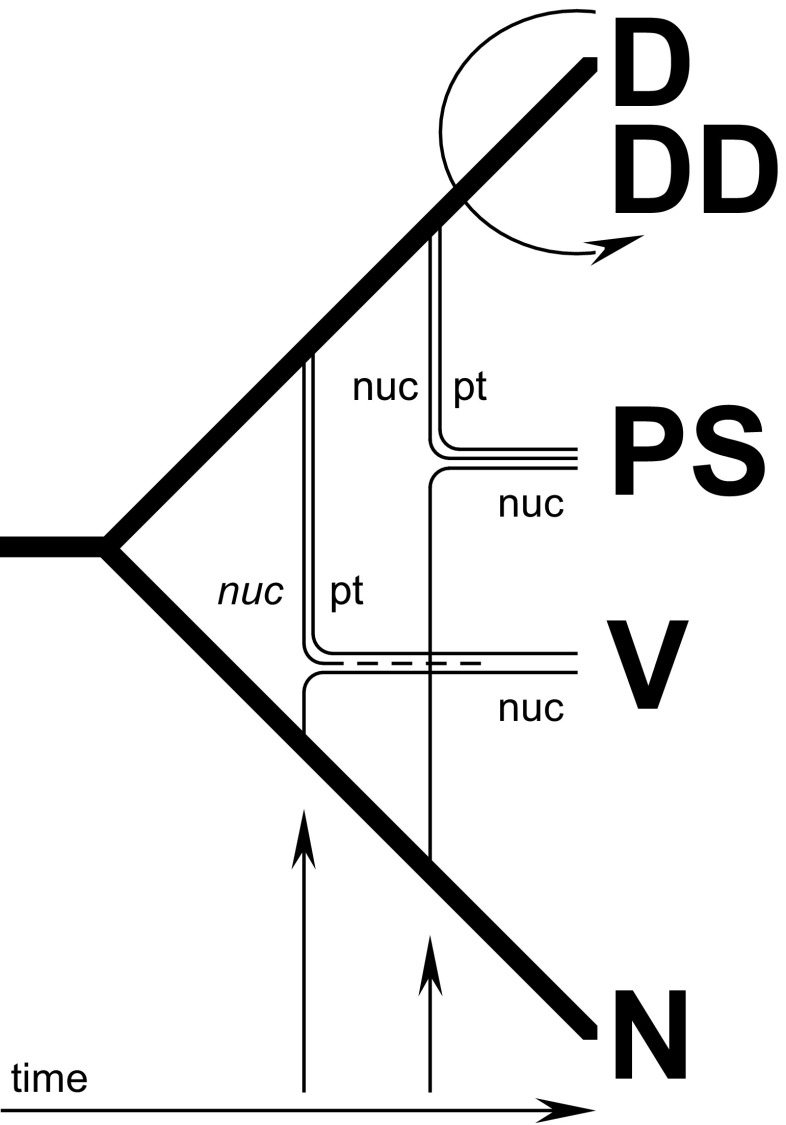
A summary model of relationships within *Cuscuta* sect. *Denticulatae* synthesized from all data presented in this study (molecular, cytological, and morphological), showing the reconstruction of cladogenesis (thick lines) and reticulation (thin lines). Hypothesized relative time frame is indicated. Abbreviations: D, *C. denticulata* (DD, autopolyploid); N, *C. nevadensis*; nuc, nuclear ribosomal arrays (biparentally inherited; lost copy shown as a dotted line); PS, *C. psorothamnensis*; pt., plastid genes (maternally inherited); V, *C. veatchii*

Figure 6 provides a schematic overview of phylogenetic relationships among species of *Cuscuta* sect. *Denticulatae*, including reconstruction of cladogenesis and reticulation in this group. The initial phylogenetic evidence on the hybrid origin of *C. veatchii* found by Stefanović and Costea (2008) is now reconfirmed and is further supported when our extensive sampling is included in the morphological and molecular analyses. Concerns about potential topological artifacts, a simple NNI topological distortion, that might be derived from a limited sampling of only three ingroup species and one outgroup are here alleviated. As previously proposed, our data support *C. denticulata* as the maternal and *C. nevadensis* as the paternal taxa. In spite of the intensive sampling and cloning, including multiple individuals of *C. veatchii*, none or very few nrITS polymorphisms were detected, suggesting a complete homogenization by concerted evolution to the rDNA type of *C. nevadensis*. The populations of *C. psorothamnensis*, described here as a new species, share morphological characteristics of *C. veatchii* (Fig. 4) but are well differentiated from it both geographically and by host specificity (Figs. 1 and 5). The direction of hybridization is the same as in *C. veatchii*, but both maternal and paternal nrDNA types are present in the genome of *C. psorothamnensis*, lacking concerted evolution. Based on the retention of both nrDNA types, it appears that this species is the result of a more recent and independent hybridization event because time has not yet been sufficient for the homogenization of the ribosomal arrays. The notion that *C. psorothamnensis* is probably a more recent allotetraploid is further supported by its geographical distribution, parapatric in a narrow zone along the southern limit of the distribution of *C. denticulata* (Fig. 1). It also grows exclusively on *P. schottii.* While this genus is known as occasional host for both diploid parentals, this particular species is not parasitized by any of them (Fig. 5).

An older origin of *C. veatchii* is supported by a combination of traits: the complete homogenization of the nrDNA arrays through concerted evolution, its clearly disjunct distribution in Baja California (Mexico) where neither of the diploid parentals currently occurs, and by host specificity restricted to *Pachycormus*, a monotypic genus also endemic to Baja California, belonging to a family (Anacardiaceae) not parasitized by the diploids. A hypothetical origin of *C. veatchii* might have occurred in Baja California Peninsula if the diploids had a wider distribution range in the past and the hybridization and whole genome duplication (WGD) had taken place somewhere along the current area of distribution of *C. veatchii* or *Pachycormus*. A host shift associated with allopolyploidy would have enabled *C. veatchii* to successfully establish in the area followed by the local extinction of the diploids. Some of the hosts frequented by the diploids, like *Larrea tridentata* or *Ambrosia dumosa*, are common in Baja California (Wiggins 1980) suggesting that other biological or ecological factors prevent the presence of the diploids in the distribution area of *C. veatchii*.

An alternative scenario is the origin of *C. veatchii* in the current area of distribution of one or both diploid progenitors and the chromosomic and genomic modifications during the allopolyploidization event allowed a host shift and the migration to the South. This sympatric formation but allopatric establishment of polyploids is frequent in angiosperms (e.g., Stuessy et al. 2004; Grundt et al. 2005; Nakagawa 2006; Lo et al. 2010). The success of newly established polyploids in populations of their diploid progenitors is not necessarily guaranteed. In the absence of uniparental reproduction (e.g., by extensive selfing or apomixis), rare polyploids may suffer from frequency-dependent disadvantages of being surrounded by closely related diploid individuals (“minority cytotype exclusion”; Levin 1975; Husband 2000; Coyne and Orr 2004). The ability to colonize environments where parental cytotypes are absent can reduce competition and enhance survivorship of neopolyploids. In the case of parasitic plants, these novel environments may be new geographic areas but also new ecological niches, manifested primarily as new hosts in these plants.

Cytological evidence of the hybrid origin of *C. veatchii* and *C. psorothamnensis* is provided by their tetraploid chromosome number, the presence of a mix of 30 small (supposedly derived from *C. denticulata*) and 30 bigger (from *C. nevadensis*) chromosomes, and interphase nuclei with chromocenters smaller than in *C. nevadensis* and the rest of the chromatin denser than in *C. denticulata*. Further research on the karyotype characterization of diploids and tetraploids is necessary to reveal patterns that are present in the diploid parentals and maintained in the allotetraploids. Preliminary results using fluorescent staining (DAPI/CMA) and fluorescent and genome in situ hybridization (FISH, GISH) confirm the presence of two sets of chromosomes in *C. veatchii*, with banding patterns and morphological features of the two diploid species (Ibiapino et al., in preparation).

### *C. denticulata* autotetraploids

The tetraploids from the Walker Pass (Kern Co., CA) that were identified morphologically as *C. denticulata* have a D1 ribotype, not N2/D2 as in *C. veatchii* and *C. psorothamnensis* (Suppl. Fig. 1 and Fig. 1, solid circles). Also, *trnL-F* sequences belong to D1 haplotype, not D2 as in the allotetraploids. In the case of a hybrid origin of these tetraploid plants, the progenitors involved would have been from different populations compared to those involved in the origin of *C. veatchii* and *C. psorothamnensis*. Even though there is no topological incongruence between nrITS and plastid sequences for these individuals, a hybrid origin cannot be dismissed because concerted evolution might have occurred, removing the *C. nevadensis* arrays and keeping only those of *C. denticulata*. However, we argue that these plants are autotetraploids and not allopolyploids, for a number of reasons: morphological, karyological, and phylogenetic (Fig. 6). Morphologically, they have very different features compared to *C. veatchii* and *C. psorothamnensis* and are instead very similar to a typical *C. denticulata* (Fig. 4), except for bigger flowers and pollen, consistent with their polyploid condition. While the chromosome number is double, the chromosome size and karyotype are uniformly similar to those of *C. denticulata* (Fig. 3). Lack of a mix of larger and smaller chromosomes, as observed in hybrid polyploids (*C. veatchii* and *C. psorothamnensis*), is an additional line of evidence for autopolyploidy in these individuals of *C. denticulata*. Tetrasomic segregation and multivalent formation are normally expected in meiosis of autopolyploids, but unfortunately, we could not observe meiosis from limited samples in hand. Up to this point, the chromosome material has been obtained only from one locality (Walker Pass, Kern Co., CA) but we have studied herbarium specimens morphologically similar from Inyo Co., CA (*Henrickson 17713*), suggesting the existence of additional autotetraploid populations, which we hope to include in our future studies. Finally, our preliminary phylogenetic results on sequencing and cloning of multiple low copy nuclear pentatricopeptide repeat (PPR) genes (García et al. in preparation) also indicate the presence of subgenomes exclusively from *C. denticulata* in these tetraploids.

The autotetraploid cytotype occurs sympatric with the diploids but might show an incipient niche separation by growing exclusively on *Ericameria* (Compositae). As for allopolyploids, the host shift may provide an advantage for newly formed polyploids by reducing competition with adjacent diploid populations through the specialization to one host which is not at all (or is much less) frequented by the diploids. Our preliminary field observations suggest also a different phenology compared to the diploids, with the tetraploids having a later flowering time. Further research is necessary to document the extent of autopolyploidy in *C. denticulata*, including the study of more individuals from this and other morphologically similar populations together with their host range.

### Future directions

Most studies about hybridization and polyploidy, including genetic and genomic aspects of these important evolutionary phenomena, are limited to a few crop and genetic model systems (Soltis et al. 2016). Few studies, however, have examined the ecological context inherent to natural systems to understand the effects of hybridization and WGD to ecology and physiology of affected plants. More studies on non-model and natural systems, in which one could begin to consider ecological effects as well, are necessary to understand the role of polyploidy in species differentiation and evolution. One such promising model is *Mimulus* (Phyrmaceae; Vallejo-Marín et al. 2015) but currently there are very few other well-documented natural systems with recurrent allopolyploidy. *Cuscuta* sect. *Denticulatae* is, to our knowledge, the first case of recurrent allopolyploidy documented for any group of parasitic plants (Figs. 2 and 6). In the root parasitic family Orobanchaceae, polyploidy is common and allopolyploidy has been proposed as the origin of a few species in the hemiparasitic genus *Castilleja* (Tank and Olmstead 2009). However, the exact parental species implicated in the formation of the allopolyploids and their cytology were not determined.

A relatively simple evolutionary model *Cuscuta* sect. *Denticulate* as described here (Fig. 6) offers the opportunity to compare expression profiles between known diploid parents and to identify post-polyploid changes in gene co-expression using two independent lineages of allopolyploids. The presence of an autotetraploid in this system would allow to take into account the dosage effects with respect to at least one (maternal) progenitor and hence tease apart the effect of allele numbers from that of homeolog gene silencing. Searching for differences in transcription between *C. veatchii* and *C. psorothamnensis* will provide important information on whether evolution repeats itself: to what extent there are differences in gene expression between recurring polyploids and are those changes predictable or stochastic? A promising line of research in this model would also be to compare specifically the haustorial gene expression between the more generalist diploids and the very host-specific allotetraploids. The allopolyploids have the genetic material of diploid species that allow them to grow on a diverse range on host species. However, hybridization and/or polyploidy have resulted in two host-specific lineages that apparently have lost the ability to grow on hosts frequented by the progenitors. This kind of comparative study has the potential to provide insights into key genes that determine host preferences in parasitic plants and improve our understanding of the genetic basis of host switch in these plants.

### Taxonomic treatment

Even though *C. psorothamnensis* has originated from the same parents as *C. veatchii*, there is a clear differentiation in ecology, host specificity, and distribution between these two species as well as with their diploid parents. Host-switching has been considered an important driver of speciation in parasitic organisms, especially in obligate parasitic plants (Schneider et al. 2016). The description of *C. psorothamnensis* will allow the easy comparison of genomic, chromosomal, transposon activation, ecological, and other data in future studies.

***Cuscuta psorothamnensis*** Stefanović, M.A. García & Costea, **sp. nov.**

**Fig. 7:**
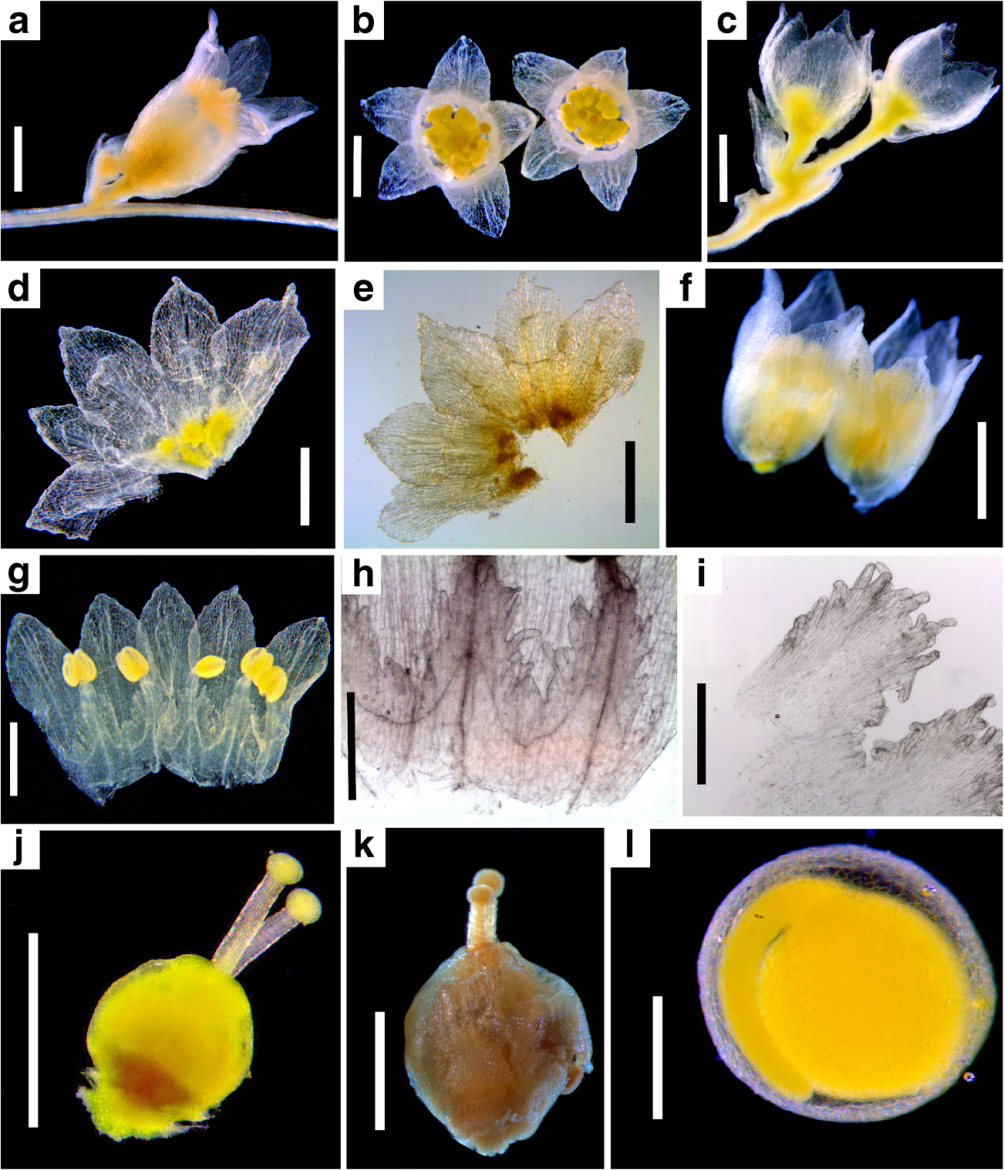
Morphology of *Cuscuta psorothamnensis*. **a** Flower, lateral view (note the axillary floral bud indicating the development of a second flower). **b** Flowers, top view. **c** Calyces after the removal of corolla. **d**, **e** Dissected calyx imaged with both diffuse (**d**) and transmitted light (**e**). **f** Corollas after the removal of calyces. **g** Dissected corolla. **h**, **i** Infrastaminal scale variation, attached to corolla tube (**h**) and detached from it (**i**). **j** Gynoecium. **k** Indehiscent capsule. **l** Embryo still surrounded by endosperm; note the globose structure characteristic to sect. *Denticulatae.* Scale bars = 1 mm except **i** which is 0.5 mm

Type: USA California; San Diego Co., Anza-Borrego Desert State Park, Hwy S2, mile 51, *Stefanović SS-13-07*, 21 April 2013 (holotype: TRTE, isotypes: WLU, NY) (Fig. 7)

*Diagnosis*: Similar morphologically to *Cuscuta veatchii* but flowers usually possess longer pedicels; calyces are glossy, straw-yellow, and the host is *Psorothamnus schottii*.

**Stems**: filiform to medium, yellow. **Inflorescences**: two to five-flowered cymose clusters or flowers solitary; bracts 1–2 at the base of pedicels and 0–1 at the base of flowers, 0.8–1.6 × 0.6–1 mm, membranous, ovate-lanceolate, margins entire to irregular, apex acute; pedicels 0.5–1.8 mm. **Flowers**: (4–)5-merous, 2.6–3 mm long, membranous; papillae absent but cells of corolla lobe tips dome-like; laticifers evident in the calyx and corolla, isolated or in rows of two to three; calyx 1.6–1.9 mm long, straw-yellow, reticulate and glossy, divided ca. 2/3 the length, tube 0.6–0.8 mm long, campanulate, as long as the corolla tube or somewhat longer, lobes 0.9–1.2 mm long, basally overlapping, ovate-triangular, not carinate, margins ± irregular or with a few teeth, apex acute; corolla 2.4–2.7 mm long, white when fresh, cream-yellow when dried, tube 1–1.2 mm long, campanulate, lobes 1–1.6 mm long initially erect later spreading to reflexed, somewhat longer than the tube, ovate, margins entire to irregular, apex acute to subacute, straight; stamens included, shorter than corolla lobes, anthers subround to broadly elliptic, 0.3–0.5 × 0.25–0.4 mm, filaments 0.2–0.4 mm long; pollen 3(-4)-zonocolpate, subprolate to prolate, 19–23 × 13–19 μm, tectum imperforate or with a few isolated punctae, sexine scabrate with isolated granules; infrastaminal scales 0.8–1.4 mm long, equaling corolla tube, bridged at 0.4–0.7 mm, ovate to oblong, truncate, with 10–16 fimbriae, 0.4–0.6 mm long; styles uniformly filiform, 0.3–0.7 mm long, 1/3–1/2 as long as the ovary; stigmas capitate, globose. **Capsules**: indehiscent, globose-ovoid to ovoid, 1.1–2 × 1–1.6 mm, not thickened or risen around the inconspicuous interstylar aperture, not translucent, surrounded by the corolla. **Seeds**: one per capsule, globose to globose-ovoid, 1–1.4 × 0.8–1.4 mm, seed coat cells alveolate/papillate, hilum region ca. 0.2 mm in diameter, terminal, sunken, scar 0.08–0.18 mm, embryo globose-enlarged toward the radicular end.

*Etymology*: The specific name stems from the name of the genus, *Psorothamnus*, to which the new species is host-specific.

*Distribution and ecology*: Flowering March–April; elevation 100–500 m; vegetation of *Larrea-Ambrosia* co-dominant with indigo bush (*P. schottii*) on bajadas, fans, and lower slopes in Anza-Borrego Desert State Park; host: *P. schottii.* For specimens examined, see Appendix 1.

### Key to the species of *Cuscuta* sect. *Denticulatae*

1. Cymes glomerulate; floral buds rounded; calyx lobes obovate-orbicular, apically rounded to obtuse, margins denticulate……………………………………*C. denticulata*

1. Cymes umbellate; floral buds acute; calyx lobes broadly ovate to triangular lanceolate, apically acute to acuminate, margins denticulate to ± entire ……...…………2

2. Cymes of 1–2(−5) flowers; flowers (2.8–)3–4(−4.5) mm long; calyx lobes triangular lanceolate, margins ± entire; corolla tube 1.3–2.2 mm long; anthers 0.5–0.8 mm long, filaments shorter than anthers………...............*C. nevadensis*

2. Cymes of (1–)2–5 flowers; flowers 2–3.2 mm long; calyx lobes oblong to broadly triangular-ovate, margins ± irregularly denticulate; corolla tube 0.8–1.6 mm long; anthers 0.3–0.5 mm long, filaments equaling anthers.......................................................3

3. Pedicels 0.5–1.2 mm; calyx dullish, brownish-yellow; corolla tube 0.8–1.6 mm long; host *Pachycormus discolor* (Anacardiaceae) …….......….…………….……*C. veatchii*

3. Pedicels 0.5–1.8 mm; calyx glossy, straw-yellow; corolla tube 1–1.2 mm long; host: *Psorothamnus schottii* (Fabaceae)…..…………*C. psorothamnensis*

### Electronic supplementary material

Table S1 (DOCX 25 kb)

Supplemental Fig. S1 Phylogenetic relationships among *Cuscuta* sect. *Denticulatae* resulting from the Bayesian analysis of all the ribosomal nuclear ITS sequences (nrITS). Upper case letters after the DNA accession numbers indicate individuals growing on different hosts in the same population. Lower case letters indicate individual seedlings from the same mother plant. Cloned accessions are indicated with “cl” followed by the clone number. Numbers above branches indicate Bayesian Posterior Probability values ≥ 0.93, whereas Parsimony Bootstrap Support values ≥ 60 are indicated below. (PNG 823 kb)

High resolution image (EPS 367 kb)

Supplemental Fig. S2 Phylogenetic relationships among *Cuscuta* sect. *Denticulatae* resulting from the Bayesian analysis of all the chloroplast *trnL-F* sequences. Upper case letters after the DNA accession numbers indicate individuals growing on different hosts in the same population. Lower case letters indicate individual seedlings from the same mother plant. Numbers above branches indicate Bayesian Posterior Probability values ≥ 0.93, whereas Parsimony Bootstrap Support values ≥ 50 are indicated below. (PNG 443 kb)

High resolution image (EPS 288 kb)

Supplemental Fig. S3 Phylogenetic relationships among *Cuscuta* sect. *Denticulatae* resulting from the Bayesian analysis of the combined nuclear and chloroplast sequences and excluding the taxa of hybrid origin. Upper case letters after the DNA accession numbers indicate individuals growing on different hosts in the same population. Lower case letters indicate individual seedlings from the same mother plant. Numbers above branches indicate Bayesian Posterior Probability values ≥ 0.93, whereas Parsimony Bootstrap Support values ≥ 50 are indicated below. (PNG 309 kb)

High resolution image (EPS 276 kb)

Supplemental Fig. S4 Phenogram resulted from the unweighted pair-group average (UPGMA) analysis using the Gower’s coefficient of similarity showing clearly delineated clusters of *C. denticulata* and *C. nevadensis*. *Cuscuta psorothamnensis* individuals clustered within *C. veatchii*. Cophenetic correlation coefficient = 0.8177. Blue head arrows indicate the specimens of *C. psorothamnensis*; green head arrows point out *SS-13-33 A, B* (2*n*=60) and the presumed autotetraploid *Henrickson 17713*. For details on the specimens used see Appendix 1. (PNG 2729 kb)

High resolution image (EPS 8163 kb)

## Appendix 1

Herbarium vouchers used for morphometric, molecular and/or cytological analyses of *Cuscuta* sect. *Denticulatae*. Country, county, locality, date, collector(s), and herbarium acronym are provided. For material used in molecular analyses, DNA extraction (as shown on phylogenetic trees) and GenBank accession numbers are given in square parentheses for *trnL-F* and ITS separated by a forward slash (/). When multiple sequences were obtained, uppercase letters indicate individuals growing on different hosts in the same population, lower case letters indicate individual or mixed seedlings from the same mother plant, and ITS clones are indicated with numbers (see Suppl. Figs. 1 and 2). A dash (−) indicates missing data. Specimens that were used both for molecular and morphometric analyses are indicated with an “asterisk.” For those vouchers analyzed cytologically, the chromosome counts are also provided in bold.

1. ***Cuscuta denticulata*****. USA Arizona.** Mohave Co.: 15 km S of Wikieup, 6 May 1993, *Baker* et al. *10732* (ASU) [#668, EF194411, EF194628]; **California.** Inyo Co.: Hwy 178, 3 mi E of Shoshone, 23 June 2013, *Stefanović SS-13-28* (seeds only); [#1478, MH920279 (a), MH920280 (mix) / MH923110 (a,b,c,d)], *Stefanović SS 13–28A, B** (TRTE, WLU), **2*****n*** **= 30**; Panamint Valley Rd. 0.5 mi W of Ballarat (Ghost town), 24 June 2013, *Stefanović SS 13–31* (seeds only) [#1479, MH920281 (a,b,c,d,mix) / MH923111 (a,b,c,d)], **2*****n*** **= 30**; Death Valley NP, Hwy 267, 16 mi E of Scott’s Castle, 28 June 2013, *Stefanović SS 13–46* (TRTE) [#1481, MH920282 (a,b,c,d,mix) / MH923112 (b,c,d)], **2*****n*** **= 30**; Surprise Canyon, 27 March 1972, *Wiggins 21769* (DS); 1 mi E of Ballarat, 12 May 1947, *Munz 11731* (SD, UCR); Steep canyon leading into Saline Valley, 16 September 1960, *Thomas 8904* (SD); Horton Creek, 15 September 1962, *Wheeler 8206* (UCR); 18 mi S of Olancha, 20 October 1978, *Henrickson 17713* (NY); 6 mi NE of Little Lake, 12 June 1979, *Henrickson 18287* (SD); Pleasant Canyon, 8 June 1995, *White & Wilcox 3251* (UCR); Kern Co.: Canebrake Valley, 7 mi E of Onyx, 9 November 1970, *Howell 47558* (QFA) [#1474, MH920277 / MH923108]; Hwy. 178, 10 and 15 mi W of Hwy. 14, Walker Pass, 24 June 2013, *Stefanović SS-13-33** (TRTE, WLU) [#1398, MH920271 (B,C) / MH923095 (A), MH923096 (B clone 1), MH923097 (B clone 2), MH923098 (B clone 3), MH923099 (B clone 4), MH923100 (B clone 5) MH923101 (B clone 6), MH923102 (C)], **2*****n*** **= 60**; Dry creek below the Goat Ranch, 26 November 1969, *Twisselmann 16344** (CAS) [#1473, MH920276 / MH923107]; Mono Co.: Hwy 120, 11 mi W of Benton, 26 Jun 2013, *Stefanović SS-13-40* (TRTE) [#1401, MH920273 (B) / MH923104 (A), MH923105 (B)]; Riverside Co.: Midland Rd. 7 mi N of Blythe, 22 April 2013, *Stefanović SS-13-10** (TRTE, WLU) [#1362, MH920263 (A,B) / MH923080 (A), MH923081 (B)], **2*****n*** **= 30**; Morongo Wash, 11 October 1932, *Wolf 4282* (SD); NW end of McCoy Mts., 1 May 1991, *Glenn 91–50* (UCR). San Bernardino Co.: Hwy. 95, 3 mi N of Vidal junction, 22 April 2013, *Stefanović SS-13-11** (TRTE, WLU) [#1363, MH920264 (A,B) / MH923082 (A), MH923083 (B)]; Hwy. 95, 10 mi N of Vidal junction, 22 April 2013, *Stefanović SS-13-12** (TRTE, WLU) [#1364, MH920265 / MH923084], **2*****n*** **= 30**; Hwy. 95, Essex, 23 April 2013, *Stefanović SS-13-16* (seeds only) [#1368, MH920266 / MH923085]; Hwy. 95, 10 mi W of Essex, 20 mi E of Amboy, 23 April 2013, *Stefanović SS-13-17* (TRTE) [#1369, MH920267 / MH923086]; Hwy. 95, 15 mi W of Essex, 15 mi E of Amboy, 23 April 2013, *Stefanović SS-13-19** (TRTE, WLU) [#1371, MH920268 (A,B) / MH923087 (A), MH923088 (B)], **2*****n*** **= 30**; Amboy Rd., N of Hwy 62 (N of Joshua Tree NP), 23 April 2013, *Stefanović SS-13-21** (TRTE, WLU) [#1373, MH920269 (A,B,C) / MH923089 (A), MH923090 (B clones 1, 5), MH923091 (B clones 2, 6), MH923092 (B clones 3, 4), MH923093 (C)], **2*****n*** **= 30**; Hwy. 66, 3 mi E of Amboy, 23 April 2013, *Stefanović SS-13-22** (TRTE, WLU) [#1374, MH920270 / MH923094], **2*****n*** **= 30**; Hwy 127, 30 mi N of Baker, 23 June 2013, *Stefanović SS-13-25* (seeds only) [#1477, MH920278 (a,b,c,d,mix) / MH923109 (a,b,c,d)], **2*****n*** **= 30**; 15 mi E from Amboy, 20 May 1920, *Munz 4181* (UCR); Whipple Mts., 16 March 1980, *Sanders 1161B* (UCR); 24 May 1980, *Sanders 1527* (UCR); E Mojave Desert, 9 June 1999, *Pitzer 3998* (UCR); 16 March 2001, Bristol Lake Basin, *Sanders 23777* (UCR); SE end of Bristol Mts., 30 May 2004, *Sanders 28080* (UCR); 4 mi NE of Amboy, 30 May 2004, *Sanders 28076* (UCR); Mojave Desert, Victor Valley, 11 June 2005, *Green s.n.* (UCR); 22 April 2011, *Sanders 39285* (UCR, WLU); 22 April 2011, *Sanders 39286* (UCR, WLU). San Diego Co.: Anza-Borrego State Park, just S of Bow Willow Campground, 32.8417 N, 116.2075 W, 10 May 2009, *Cain & Clayton 1113* (SD); Borrego Valley, 7 April 1940, *Howe 991* (SD). Tulare Co.: Lamont Meadow, *Howell 43513* (CAS). **Nevada.** Esmeralda Co.: Intersection of Hwys. 264 / 6, 25 June 2013, *Stefanović SS-13-39* (seeds only) [#1400, MH920272 / MH923103]; Washoe Co.: Pyramid Lake, Hwy. 448, 8 mi W of Nixon, 20 July 2014, *Stefanović SS-13-45** (TRTE) [#1405, MH920274 / MH923106]; Humboldt Co.: Calico Mts., foothill area W of main Gerlach-Soldier Meadow Rd., 6 July 2000, *Tiehm 13319** (ASU) [#485, EF194410 / EF194627]; NNW of Bog Hot Ranch, 12 August 1978, *Tiehm & Rogers 4724* (NY) [#1472, MH920275, −]. **Utah.** Grand Co.: E side of Ida Gulch, SW of Professor Valley, 21 September 1985, *Franklin 2601* (NY) [#1144, MH920262, MH923079]; St. George, 1874, *Parry 205* (Isotype, NY). **Washington.** Franklin Co.: Hanford Site, Wahluke Wildlife Area, White Bluffs, 19 July 1994, *Beck & Caplow 94051* (WTU) [#165, EF194409 / EF194626].

2. ***Cuscuta nevadensis*****. USA California.** Inyo Co.: Westgard Pass, 8.5 mile W of White Mt. road, 18 June 1963, *Lloyd 2639* (NY, UC) [#1145, MH920283 / MH923113]; Death Valley NP, Wildrose campground, 13 April 1968, *Pinkava* et al. *12181* (ASU) [#476, EF194407 / EF194629]; Hwy. 127, 45 mi N of Baker, 23 June 2013, *Stefanović SS-13-26** (TRTE, WLU) [#1426, MH920291 (A) / MH923132 (A, C)]; Hwy. 178, 1 mile E of Shoshone, 23 June 2013, *Stefanović SS-13-27** (TRTE, WLU) [#1396, MH920284 (B) / MH923114 (A), MH923115 (B), MH923116 (B clones 1,5), MH923117 (clone 2), MH923118 (clone 3), MH923119 (B clone 4)], **2*****n*** **c. 30**; Hwy. 190, 6 and 7.5 mi W of Death Valley Jct., 23 June 2013, *Stefanović SS-13-29** (TRTE, WLU) [#1397, MH920285 (A,B) / MH923120 (A), MH923121 (B)], **2*****n*** **= 30**; Panamint Springs, on Hwy. 190, Death Valley N.P., 23 June 2013, *Stefanović SS-13-30* (TRTE, WLU) [#1409, MH920290 / MH923131], **2*****n*** **= 30**; Hwy. 395, Coso Junction (and all along the hwy divide), 24 June 2013, *Stefanović SS-13-34* (TRTE) [#1429, MH920294 / MH923134], **2*****n*** **c. 30**; Hwy. 190, 3.5 mi NE of Olancha, 24 June 2013, *Stefanović SS-13-35** (TRTE, WLU) [#1399, MH920286 (B,C) / MH923122 (B,C)], **2*****n*** **c. 30**; Hwy. 190, 3.5 mi NE of Olancha, 25 June 2013, *Stefanović SS-13-36* (only seeds) [#1480, MH920295 (a,b,c,d,mix) / MH923135 (a,b,c,d)]; Hwy. 127, 5 mi N of Death Valley Junction, 29 June 2013, *Stefanović SS-13-48** (TRTE, WLU) [#1407, MH920289 / MH923130]; San Bernardino Co.: Hwy. 178, 17 mi S-SW of Trona, 24 June 2013, *Stefanović SS-13-32* (seeds only) [#1427, MH920292 (a), MH920293 (b,d,e,mix) / MH923133], **2*****n*** **= 30**; 8 mi W of Death Valley Junction, 4 May 1958, *Raven 12865* (CAS); *Howell 32746* (RSA); Panamint Mts., 9 July 1974, *Thorne* et al. *44889* (SD); ca. 16 mi S-SE of Olancha, 19 October 1978, *Henrickson 17659a* (NY); Ridge top NW of Indian Pass, 1 June 1983, *Peterson 888* (NY); Slate Range, above the Gold Button Mine, 8 May 1981, *Sanders 2094* (UCR); White Mts., 18 June 1984, *Morefield 2119s** (NY) [#585, EF194408 / EF194630]; Slate Range, 8 May 1981, *Sanders* et al. *2091* (UCR); Kern Co.: 6 mi E of Inyokern, 10 July 1945, *Wheeler 6087* (UCR). 3.5 mi SW of Trona, 1 July 1983, *Thorne* et al. *56068* (SD). **Nevada.** Nye Co.: Hwy. 373, intersection with Rd. to Ash Meadows, 29 June 2013, *Stefanović SS-13-47** (TRTE, WLU) [#1406, MH920287 (B,C,D), MH920288 (E) / MH923123 (B), MH923124 (E clone 1), MH923125 (E clone 2), MH923126 (E clone 3), MH923127 (E clone 4), MH923128 (E clone 5), MH923129 (E clone 6)], **2*****n*** **= 30**; Amargosa, 19 June 1969, *Beatley 9055* (RSA); Steward Valley, 12 April 1970, *Beatley 9954* (RSA).

3. ***Cuscuta psorothamnensis*****. USA California.** Imperial Co.: Near I-8 in sandy flat at mouth of In-Ko-Pah Gorge, Devil’s Canyon along Myer Creek, 17 April 1981, *Yatskievych 81–119* (ARIZ) [#555, MH920300 / MH923168 (clone 1), MH923169 (clones 2,3), MH923170 (clone 4), MH923171 (clone 5), MH923172 (clone 6), MH923173 (clone 7)]; San Diego Co.: Anza-Borrego Desert State Park, 3.2 air mi SW of Hwy 78/Split Mt. Rd., 3 March 2010, *Hendrickson* et al. *4502** (SD) [#1510, MH920297, −]; Lois Neyenesch Folly private property, just NW of Anza-Borrego Desert State Park boundary, 1 mile W of Hwy S2 at Cranebrake Canyon Rd., 32.8972° N, 116.2422° W, 20 March 2005, *Nenow and Glacy 162** (SD) [#1512, MH920299 / MH923162 (clone 1), MH923163 (clone 2), MH923164 (clone 3), MH923165 (clone 4), MH923166 (clone 5), MH923167 (clone 6)]; Anza-Borrego State Park, June Wash, 32.9775° N, 116.2475° W, 5 March 2005, *Angel 111* (SD); Anza-Borrego State Park, along Rd. S2, mile 51 and 52, 21 April 2013, *Stefanović SS-13-07** (TRTE, WLU) [#1361, MH920296 (A,B,C) / MH923136 (A clone 1), MH923137 (A clone 3), MH923138 (A clone 5), MH923139 (A clone 6), MH923140 (A clone 7), MH923141 (A clone 8), MH923142 (B clone 1), MH923143 (B clone 2), MH923144 (B clone 3), MH923145 (B clone 4), MH923146 (B clone 5), MH923147 (B clone 6), MH923148 (B clone 7), MH923149 (B clone 8), MH923150 (C clone 1), MH923151 (C clone 2), MH923152 (C clone 3), MH923153 (C clone 4), MH923154 (C clone 5), MH923155 (C clone 6), MH923156 (C clone 7)], **2*****n*** **= 60**; Anza-Borrego Desert State Park, 3.85 ESE of Hwy. 78/Old Kane Spring Rd., 33.1302° N, 116.204° W, 11 March 2009, *Sullivan 641** (SD) [#1511, MH920298 / MH923157 (clone 1), MH923158 (clone 2), MH923159 (clone 3), MH923160 (clone 4), MH923161 (clone 5)]; Hwy S2, mi 53, 6 mi N of Ocotillo, 4 April 2016, *Stefanović SS-16-12* (TRTE), **2*****n*** **= 60**; Hwy S2, mi 47, Mountain Palms Rd., 4 April 2016, *Stefanović SS-16-14* (TRTE), **2*****n*** **= 60**.
4. ***Cuscuta veatchii*****. Mexico. Baja California.** Close to Bahía de Los Ángeles, 28° 58′ 47.1″ N, 113° 43′ 13.0″ W, 298 m; 30 April 2014, *Costea s.n.** A, B (WLU) [#1463, MH920303 (A,B,C,D,E) / MH923176 (A,B,C,E), MH923177 (D clone 1), MH923178 (D clone 2), MH923179 (D clone 3), MH923180 (D clone 4), MH923181 (D clone 5)], **2*****n*****=60**; N of Punta Prieta, 30 April 2014, 28° 57′ 25.4″ N, 114° 09′ 40.2″ W, 240 m, 30 April 2014, *Costea s.n.** A, B (WLU) [#1464, MH920304 (A,B,C,D,E) / MH923182 (A,B,C,D,E)], **2*****n*** **= 60**; Punta Prieta, 11 October 1941, *Harbison 6901* (SD); 1.2 mi NW of Punta Prieta, 1 June 1973, *Spellenberg* et al. *s.n.* (NY); Close to Bajia de Los Ángeles, Arroyo El Palmarito, ca. 4.5 km NW of Cataviña, 6 June 1984, *Dice 477* (SD); Los Ángeles Bay, 5 May 1921, *Johnston 3439* (GH); 6 May 1921, Los Ángeles Bay, *Johnston 3430* (KEW); 31 July 1966, *Henrickson 2323* (MICH) (RSA*) [#580, EU288339, EU288354]; Ca. 23 mi NW of Bahia de Los Ángeles, 18 February 1979, *Devine 508* (CHSC); Cataviña Sur, ca. 60 km S of Cataviña, 30 April 2014, *Costea s.n.** A, B, C, D (WLU) [#1465, MH920305 (A,B,C,D,E) / MH923183 (A,B,C,D,E)], **2*****n*** **= 60**; Cataviña Norte, ca. 5 km N of Cataviña, 30 April 2014, *Costea s.n.** A, B, C, D (WLU) [#1466, MH920306 (A,B,C,D,E) / MH923184 (A,B,C,D,E)], **2*****n*** **= 60**; Vizcaino Desert, ca. 11 mi S of Hwy. 1, along the Rd. to Santa Catarina, 29 May 1987, *Thorne* et al. *62616* (F) [#521, EU288338 / EU288353] (NY) [#760, EU288340 / EU288355]; 5 mi N of Misión San Borjas, 17 May 1959, *Wiggins & Wiggins, 14856* (GH, WTU) [#559 = #1146, MH920301 / MH923174]; 1 mile S of Las Arrastras, 25 March 1960, *Wiggins & Wiggins, 15933* (WTU) [#1147, MH920302 / MH923175]; About 11 miles S of Hw1 along road to Cataviña, 29 May 1987, *Thorne 62616* (F); Sierra San Borja, 25 May 1996, *Rebman 3189* (SD)

## Appendix 2.

1. Pedicel length (mm); 2. Flower length (measured from the base of receptacle to tips of straightened corolla lobes, mm); 3. Calyx lobes length (mm); 4. Calyx lobes width (mm); 5. Calyx lobes, width of membranous margin (mm); 6. Calyx tube length (mm); 7. Number of teeth per calyx lobe; 8. Width of overlapping area between calyx lobes (mm); 9. Length of overlapping area between calyx lobes (mm); 10. Angle formed by margins of calyx lobes at the tips (degrees); 11. Calyx area (mm^2^); 12. Corolla tube length (mm); 13. Corolla lobes length (mm); 14. Corolla lobes width (mm); 15. Angle formed by corolla lobe margins at the tip (degrees); 16. Corolla area (mm^2^); 17. Stamen filaments length (mm); 18. Anthers length (mm); 19. Anthers width (mm); 20. Infrastaminal scale length (mm); 21. Infrastaminal scales width (mm); 22. Infrastaminal scales, bridge height (mm); 23. Infrastaminal scales, length of longest fimbriae (mm); 24. Infrastaminal scales, width of fimbriae (mm); 25. Number of fimbriae per infrastaminal scale; 26. Length of shortest style (mm); 28*. Receptacle fleshy gelatinous at the base, present (1), absent (0); 29*. Corolla: detaches easily from the receptacle/calyx (1), corolla difficult to separate from the receptacle/calyx (0); 30*. Corolla lobes margin: serrulate or denticulate (1), entire to wavy (0); 31. Pollen grains length (μm); 32. Pollen grains width (μm).
