# Supplementary material for: Cladogenesis and reticulation in Cuscuta sect. Denticulatae (Convolvulaceae)
Source: Org Divers Evol. 2018 Oct 28;18(4):383–98. doi: 10.1007/s13127-018-0383-5 (PMC6405177; doi:10.1007/s13127-018-0383-5)
Supplement: Supplementary file 1 — (DOCX 25 kb) [file 13127_2018_383_MOESM1_ESM.docx]

Cladogenesis and reticulation in *Cuscuta* sect. *Denticulatae* (Convolvulaceae)

Organisms Diversity & Evolution

Miguel A. García*, Saša Stefanović, Catherine Weiner, Magdalena Olszewski and Mihai Costea

*Corresponding author. Department of Biology, University of Toronto Mississauga, Mississauga, Ontario L5L 1C6, Canada. Royal Botanic Gardens Kew, Richmond, Surrey, TW9 3AE, United Kingdom (current address). E-mail address: ma.garcia@utoronto.ca

**Online Supplementary Table**

**Table S1**. Host range of *Cuscuta* species in sect. *Denticulatae*. Host frequency (%) is indicated for each host species. Shared host species are indicated in a gray-shaded box. Shared host genera (*Psorothamnus*) are indicated with a zig-zagged line. Data was used to create the bipartite network in Fig. 5.

| **Host species/*Cuscuta* sp.** | *C. denticulata* | *C. nevadensis* | *C. psorothamnensis* | *C. veatchii* | |
| --- | --- | --- | --- | --- | --- |
| **Anacardiaceae**  *Pachycormus discolor* | 0 | 0 | 0 | 100 |  |
| **Asteraceae** |  |  |  |  |  |
| *Acamptoppapus sphaerocephalus* | 1.86 | 2.22 | 0 | 0 |  |
| *Ambrosia dumosa* | 3.73 | 17.77 | 0 | 0 |  |
| *Artemisia ludoviciana* | 0.93 | 0 | 0 | 0 |  |
| *Artemisia tridentata* | 6.54 | 0 | 0 | 0 |  |
| *Bebbia juncea* | 0.93 | 0 | 0 | 0 |  |
| *Chrysothamnus sp.* | 6.54 | 0 | 0 | 0 |  |
| *Chrysothamnus albidus* | 0.93 | 0 | 0 | 0 |  |
| *Chrysothamnus paniculatus* | 5.6 | 0 | 0 | 0 |  |
| *Chrysothamnus nauseosus* | 9.34 | 0 | 0 | 0 |  |
| *Chrysothamnus viscidiflorus* | 4.67 | 0 | 0 | 0 |  |
| *Ericameria cooperi* | 1.86 | 0 | 0 | 0 |  |
| *Ericameria nana* | 0.93 | 0 | 0 | 0 |  |
| *Hymenoclea salsola* | 0.93 | 0 | 0 | 0 |  |
| *Lepidospartum squamulatum* | 1.86 | 0 | 0 | 0 |  |
| *Perityle emoryi* | 0.93 | 0 | 0 | 0 |  |
| *Peucephyllum schottii* | 0.93 | 0 | 0 | 0 |  |
| *Pleurocornis pluriseta* | 0.93 | 0 | 0 | 0 |  |
| *Stephanomeria* *sp.* | 0 | 4.44 | 0 | 0 |  |
| *Tetradymia sp.* | 0.93 | 0 | 0 | 0 |  |
| **Chenopodiaceae** |  |  |  |  |  |
| *Atriplex confertifolia* | 0 | 37.77 | 0 | 0 |  |
| *Atriplex hymenolytra* | 0 | 2.22 | 0 | 0 |  |
| *Atriplex polycarpa* | 0 | 4.48 | 0 | 0 |  |
| *Grayia spinosa* | 0.96 | 0 | 0 | 0 |  |
| *Suaeda moquinii* | 0.93 | 4.44 | 0 | 0 |  |
| **Brassicaceae** |  |  |  |  |  |
| *Brassica tournefortii* | 0.93 | 2.36 | 0 | 0 |  |
| *Stanleya pinnata* | 0 | 2.22 | 0 | 0 |  |
| **Euphorbiaceae** |  |  |  |  |  |
| *Euphorbia sp.* | 1.86 | 0 | 0 | 0 |  |
| *Euphorbia alnomarginata* | 0.93 | 0 | 0 | 0 |  |
| *E. polycarpa* | 1.86 | 0 | 0 | 0 |  |
| **Fabaceae** |  |  |  |  |  |
| *Psorothamnus arborescens* | 0.93 | 2.22 | 0 | 0 |  |
| *Psorothamnus spinosus* | 0.93 | 2.41 | 0 | 0 |  |
| *Psorothamnus fremontii* | 0 | 11.11 | 0 | 0 |  |
| *Psorothamnus emoryi* | 0.81 | 0 | 0 | 0 |  |
| *Psorothamnus schotii* | 0 | 0 | 100 | 0 |  |
| **Lamiaceae** |  |  |  |  |  |
| *Scutellaria austineae* | 0.84 | 0 | 0 | 0 |  |
| **Plantaginaceae** |  |  |  |  |  |
| *Plantago* | 0.93 | 0 | 0 | 0 |  |
| **Polygonaceae** |  |  |  |  |  |
| *Dedeckera eurekensis* | 0 | 2.48 | 0 | 0 |  |
| *Eriogonum contortum* | 0.93 | 0 | 0 | 0 |  |
| *E. inflatum* | 0.84 | 0 | 0 | 0 |  |
| *E. fasciculatum* | 0.96 | 0 | 0 | 0 |  |
| **Rosaceae** |  |  |  |  |  |
| *Coleogyne ramosissima* | 1.86 | 0 | 0 | 0 |  |
| **Solanaceae** |  |  |  |  |  |
| *Lycium sp.* | 1.86 | 0 | 0 | 0 |  |
| *Lycium andersonii* | 0.93 | 2.22 | 0 | 0 |  |
| **Zygophyllaceae** |  |  |  |  |  |
| *Covillea sp.* | 0.93 | 0 | 0 | 0 |  |
| *Larrea tridentata* | 28.97 | 0 | 0 | 0 |  |
