# Supplementary figures and images for: Cladogenesis and reticulation in Cuscuta sect. Denticulatae (Convolvulaceae)
Source: Org Divers Evol. 2018 Oct 28;18(4):383–98. doi: 10.1007/s13127-018-0383-5 (PMC6405177; doi:10.1007/s13127-018-0383-5)

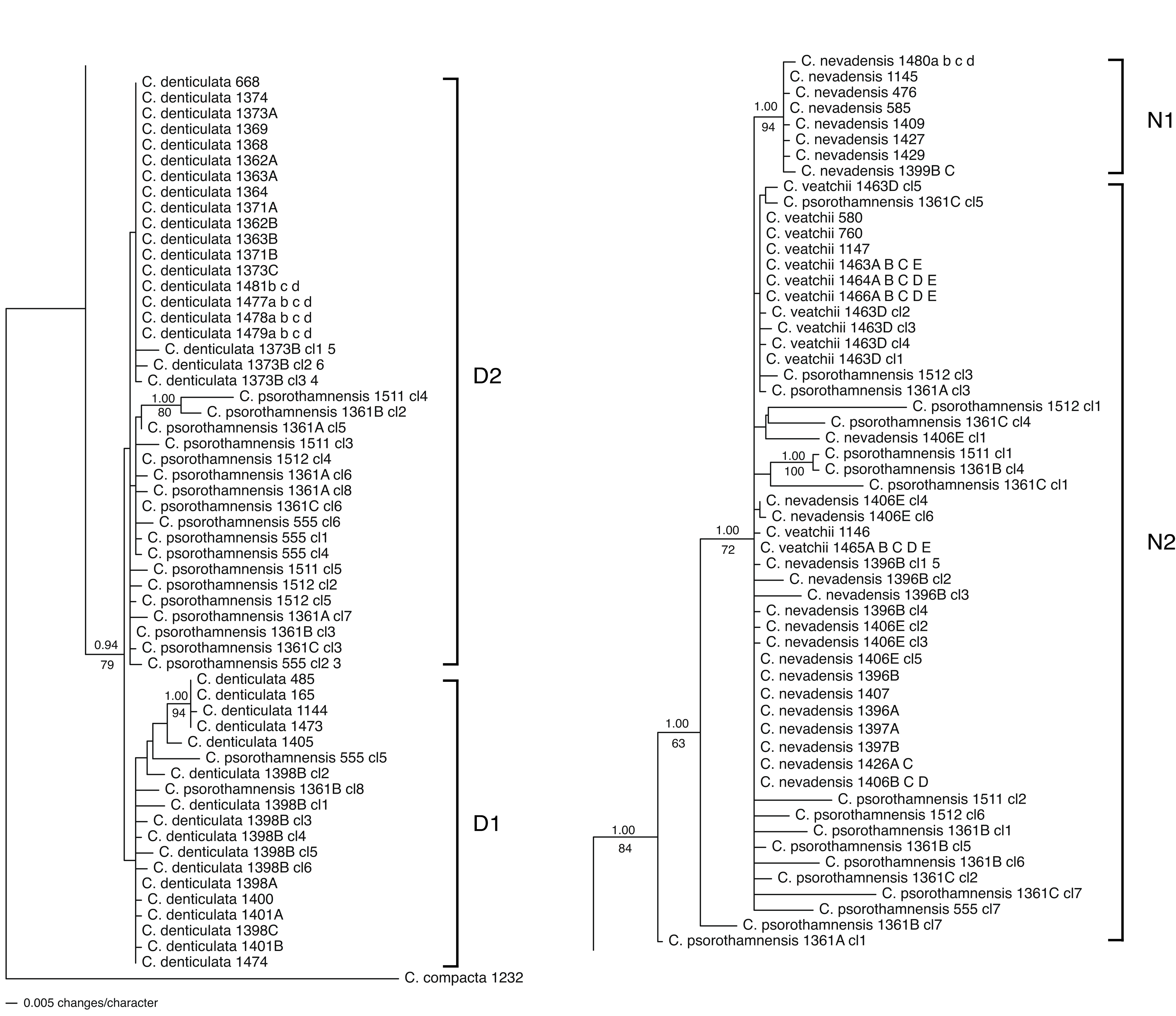

Supplement: Supplementary file 2 — Phylogenetic relationships among Cuscuta sect. Denticulatae resulting from the Bayesian analysis of all the ribosomal nuclear ITS sequences (nrITS). Upper case letters after the DNA accession numbers indicate individuals growing on different hosts in the same population. Lower case letters indicate individual seedlings from the same mother plant. Cloned accessions are indicated with “cl” followed by the clone number. Numbers above branches indicate Bayesian Posterior Probability values ≥ 0.93, whereas Parsimony Bootstrap Support values ≥ 60 are indicated below. (PNG 823 kb) [file 13127_2018_383_Fig8_ESM.png]

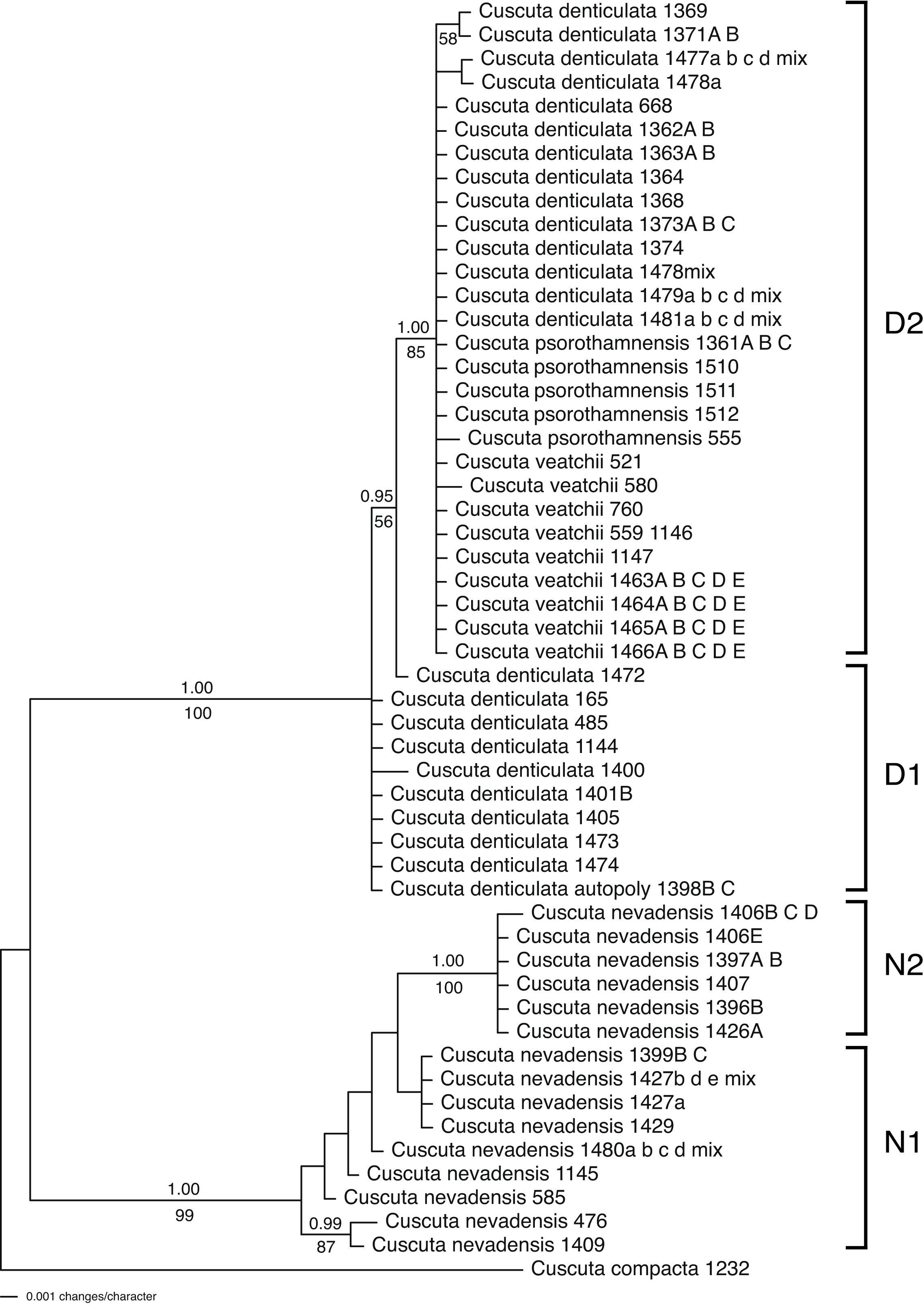

Supplement: Supplementary file 4 — Phylogenetic relationships among Cuscuta sect. Denticulatae resulting from the Bayesian analysis of all the chloroplast trnL-F sequences. Upper case letters after the DNA accession numbers indicate individuals growing on different hosts in the same population. Lower case letters indicate individual seedlings from the same mother plant. Numbers above branches indicate Bayesian Posterior Probability values ≥ 0.93, whereas Parsimony Bootstrap Support values ≥ 50 are indicated below. (PNG 443 kb) [file 13127_2018_383_Fig9_ESM.png]

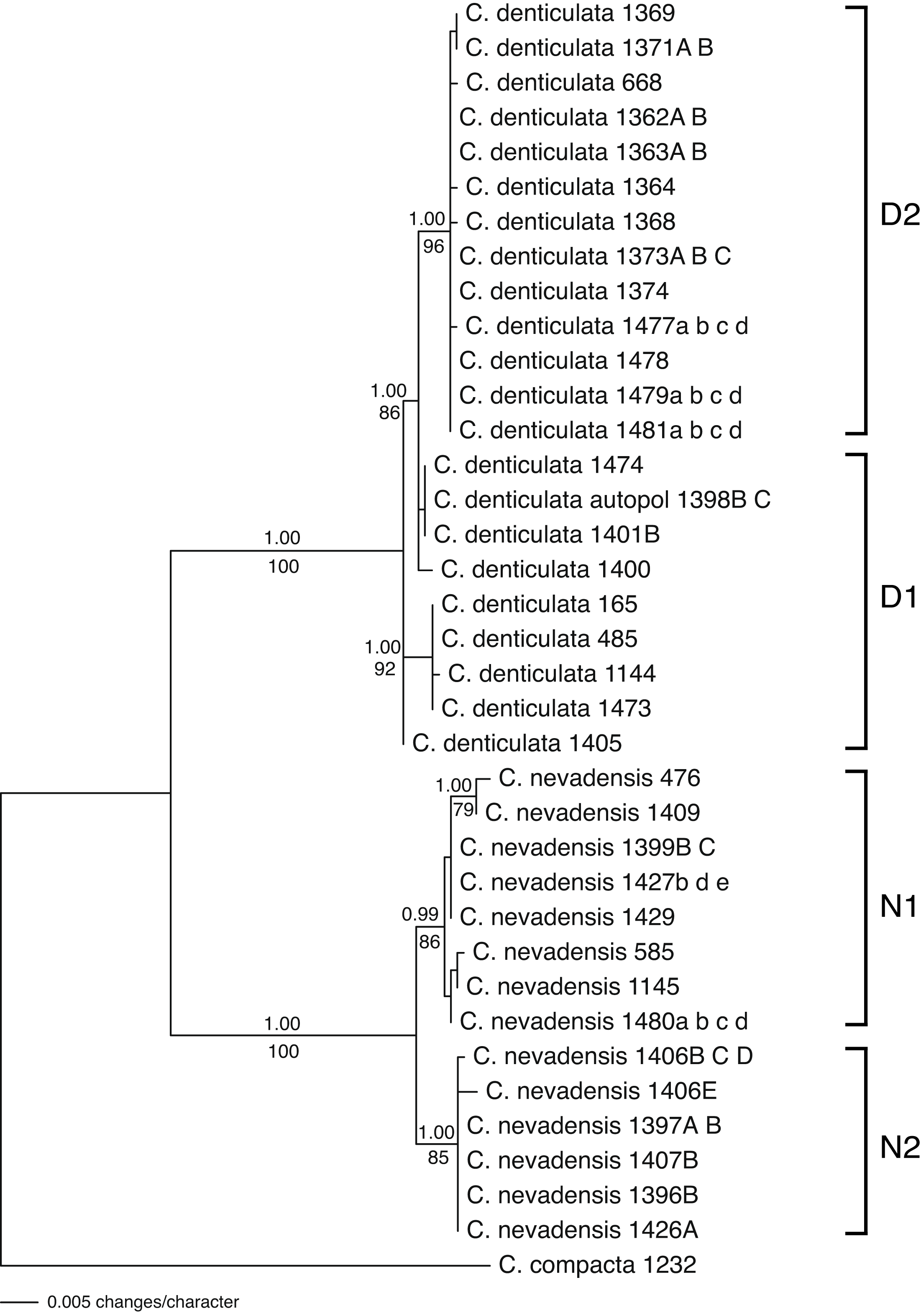

Supplement: Supplementary file 6 — Phylogenetic relationships among Cuscuta sect. Denticulatae resulting from the Bayesian analysis of the combined nuclear and chloroplast sequences and excluding the taxa of hybrid origin. Upper case letters after the DNA accession numbers indicate individuals growing on different hosts in the same population. Lower case letters indicate individual seedlings from the same mother plant. Numbers above branches indicate Bayesian Posterior Probability values ≥ 0.93, whereas Parsimony Bootstrap Support values ≥ 50 are indicated below. (PNG 309 kb) [file 13127_2018_383_Fig10_ESM.png]

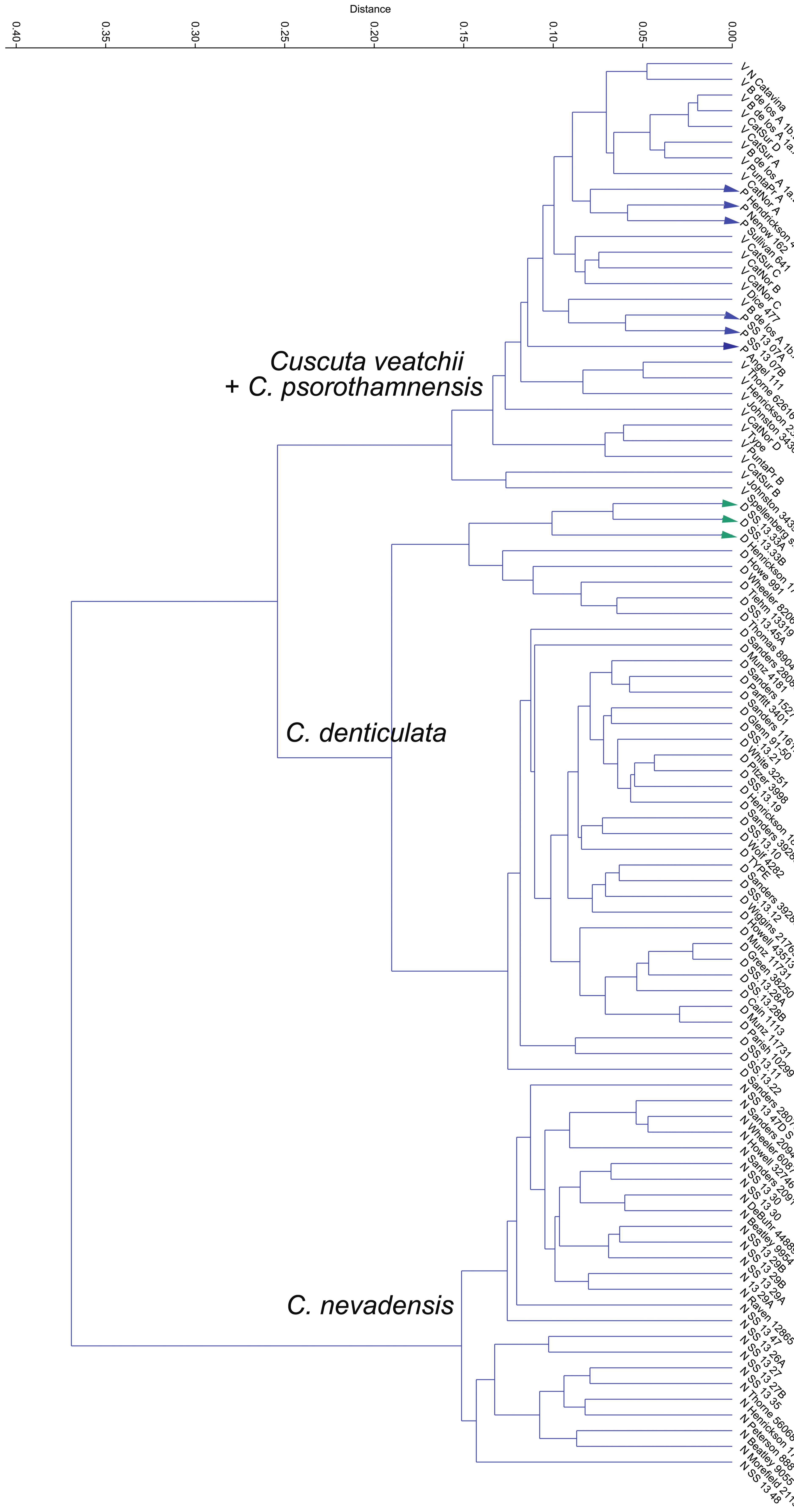

Supplement: Supplementary file 8 — Phenogram resulted from the unweighted pair-group average (UPGMA) analysis using the Gower’s coefficient of similarity showing clearly delineated clusters of C. denticulata and C. nevadensis. Cuscuta psorothamnensis individuals clustered within C. veatchii. Cophenetic correlation coefficient = 0.8177. Blue head arrows indicate the specimens of C. psorothamnensis; green head arrows point out SS-13-33 A, B (2n=60) and the presumed autotetraploid Henrickson 17713. For details on the specimens used see Appendix 1. (PNG 2729 kb) [file 13127_2018_383_Fig11_ESM.png]
